# Comparative effectiveness of dietary supplements in dilated cardiomyopathy: a systematic review and network meta-analysis

**DOI:** 10.1186/s12872-026-06509-x

**Published:** 2026-08-27

**Authors:** Yiying Liu, Ruikang Liu, Jun Li, Kai Yang, Yongmei Liu, Fuyuan Zhang, Guancheng Ye, Yulian Yuan

**Affiliations:** 1 Department of Cardiology, Guang’anmen Hospital, China Academy of Chinese Medical Sciences, Xicheng District, Beijing, 100053 China; 2 Graduate School, Beijing University of Chinese Medicine, Chaoyang District, Beijing, 100029 China; 3 Department of Rheumatology, Dongzhimen Hospital, Beijing University of Chinese Medicine, Beijing, 100700 China; 4 School of Medicine, Tsinghua University, Haidian District, Beijing, 100084 China; 5 Yuquan Hospital, Tsinghua University, Shijingshan District, Beijing, 100049 China

**Keywords:** Dilated cardiomyopathy, Dietary supplements, Coenzyme Q_10_, Taurine, n-3 polyunsaturated fatty acids, Network meta-analysis

## Abstract

**Background:**

Dilated cardiomyopathy (DCM) is a major cause of heart failure and cardiovascular mortality, partly due to impaired myocardial energy metabolism. Dietary supplements may offer benefits through improved mitochondrial function and antioxidant effects, and randomized controlled trials (RCTs) suggest they can enhance cardiac function in DCM. However, direct comparisons among supplements are lacking, and the optimal strategy and dose–response relationship remain undetermined.

**Methods:**

Eight databases were searched for RCTs evaluating dietary supplements in DCM. Continuous outcomes were analysed by frequentist random-effects network meta-analysis (NMA; Stata/MP 18.0); N-terminal pro-B-type natriuretic peptide (NT-proBNP) and New York Heart Association (NYHA) class were synthesised by pairwise meta-analysis. Risk of bias was assessed with the Cochrane Risk-of-Bias 2 (RoB 2) tool and certainty of evidence with the Grading of Recommendations Assessment, Development and Evaluation (GRADE) approach.

**Results:**

Fourteen RCTs (1,259 participants) were included. For left ventricular ejection fraction (LVEF), coenzyme Q_10_ (CoQ_10_) 150 mg/d produced the largest improvement versus conventional treatment (CT) (mean difference [MD] = 11.26, 95% confidence interval [CI] 8.55 ~ 13.98; surface under the cumulative ranking curve [SUCRA] 93.4%), followed by taurine and CoQ_10_ 90 mg/d. CoQ_10_ 150 mg/d also ranked first for reducing left ventricular end-diastolic diameter (LVEDD; −5.40, − 8.14 ~ − 2.66) and end-systolic diameter (LVESD; −6.31, − 8.60 ~ − 4.01). For heart rate, n-3 polyunsaturated fatty acids (n-3 PUFAs) gave the most robust reduction (− 3.94 bpm, − 5.11 ~ − 2.77). In pairwise meta-analysis, CoQ_10_ lowered NT-proBNP and improved NYHA status (risk ratio [RR] for ≥ 1-class improvement 1.23, 1.10 ~ 1.38). Network inconsistency was significant for LVESD (*P* = 0.008). Certainty of evidence (GRADE) was low to very low, and moderate only for n-3 PUFAs; all supplements were well tolerated.

**Conclusions:**

In this network, higher-dose CoQ_10_ (150 mg/d) showed the most favourable overall profile for systolic function and ventricular dimensions, while n-3 PUFAs offered the most reliable benefit for heart rate. Given small samples, predominantly Chinese open-label trials, a heterogeneous and time-varying CT comparator, local inconsistency, and poor safety reporting, these findings should be regarded as hypothesis-generating and require confirmation in larger, well-designed international RCTs with longer follow-up.

**Supplementary Information:**

The online version contains supplementary material available at https://doi.org/10.1186/s12872-026-06509-x.

Dilated cardiomyopathy (DCM) significantly contributes to global heart failure and cardiovascular mortality. It involves progressive dilation of the left or both ventricles with reduced contractile function, excluding abnormal workload and significant coronary artery disease as causes of global systolic dysfunction [1, 2]. This disease greatly diminishes patients’ quality of life and is linked to serious cardiovascular complications such as sudden cardiac death, thromboembolic events, and progressive heart failure [3]. Epidemiological data show that DCM ranks as the third leading cause of heart failure and is a main reason for heart transplantation, with an in-hospital mortality rate reaching 8.7%. Sudden cardiac death can be the first clinical sign, occurring annually in about 2%–4% of cases [4–6].

The development of DCM arises from the interplay between genetic predispositions and multiple environmental or acquired factors [7], such as genetic variants, viral infections, autoimmune issues, toxic exposures, and metabolic disturbances [8, 9]. A central aspect of disease progression lies in the disruption of myocardial energy metabolism, specifically mitochondrial dysfunction, increased oxidative stress, and impaired adenosine triphosphate (ATP) synthesis [10]. Reduced activity of the mitochondrial electron transport chain and impaired antioxidant defence can induce contractile dysfunction and promote ventricular remodeling [11]. High reactive oxygen species (ROS) levels can intensify myocardial damage via lipid peroxidation, protein oxidation, and mitochondrial DNA damage [12].

Current standard pharmacological treatments for DCM primarily target neurohormonal activation and symptom relief, without directly intervening in metabolic or oxidative-stress pathways [13, 14]. Adjunctive strategies aimed at restoring myocardial energy metabolism therefore address an important gap. Recent RCTs indicate that dietary supplements such as coenzyme Q_10_ (CoQ_10_), taurine, and n-3 polyunsaturated fatty acids (n-3 PUFAs) enhance cardiac function and associated biomarkers in DCM patients. Most studies, however, assessed these interventions in isolation or in placebo-controlled designs, which do not adequately address comparative efficacy and optimal dosing.

Network meta-analysis (NMA) enhances traditional meta-analysis by combining direct and indirect evidence, allowing concurrent assessment of multiple interventions [15–18]. To our knowledge, no NMA has specifically compared the efficacy of dietary supplements in the DCM population. This study evaluates the efficacy and safety of dietary supplements in modulating left ventricular ejection fraction (LVEF), ventricular dimensions, neurohormonal markers, and functional status in DCM patients.

## Materials and methods

### Search strategy

A comprehensive literature search was performed across eight databases: PubMed, Cochrane Library, Embase, Web of Science, CNKI, Wanfang, VIP, and SinoMed, using automated and manual methods, and updated through February 15, 2026. Two independent reviewers (Y.L. and R.L.) conducted screening and data extraction; discrepancies were resolved by a third reviewer (K.Y.). All three reviewers are medically trained clinician-researchers specialising in cardiovascular medicine / traditional Chinese medicine cardiology and each has more than five years of experience in evidence-based medicine and systematic-review research. Before formal screening, they received internal training on the PRISMA 2020 workflow and dual-review procedures and piloted the eligibility form on a sample of records to calibrate decisions. RCTs published in Chinese and English were eligible. Study selection followed the Preferred Reporting Items for Systematic Reviews and Meta-Analyses (PRISMA) 2020 guidelines [19], and the protocol was registered with PROSPERO (CRD420261351179). Full search strings are provided in Appendix S1.

### Inclusion and exclusion criteria

Eligibility followed the Population, Intervention, Comparator, Outcomes, Study design (PICOS) framework (Table 1). Eligible studies were RCTs of adults (≥ 18 years) with a guideline-based diagnosis of DCM (reduced LVEF and ventricular dilation). The treatment arm received a dietary supplement; the comparator was conventional treatment (CT), placebo, or no additional intervention. At least one of the following outcomes was required: LVEF, left ventricular end-diastolic diameter (LVEDD), left ventricular end-systolic diameter (LVESD), N-terminal pro-B-type natriuretic peptide (NT-proBNP), New York Heart Association (NYHA) class, or heart rate (HR). Candidate supplements were identified by systematically searching for substances with a plausible mechanistic rationale for improving myocardial energy metabolism, oxidative stress, or calcium handling in DCM (coenzyme Q_10_, taurine, n-3 PUFAs, L-carnitine, creatine, thiamine, vitamin C, vitamin D, and selenium). Supplements were eligible for inclusion only if supported by at least one RCT reporting cardiac function or structure outcomes in adult DCM. Nitrates were classified as conventional therapy rather than dietary supplementation. CoQ_10_ was categorised as 30, 60, 90, or 150 mg/d according to the reported regimen; when a reported dose range spanned categories, the maximum protocol daily dose determined the network node.

**Table 1:** PICOS criteria

Criteria	Explanation
Population	Adults (≥ 18 years) with a guideline-based diagnosis of DCM (reduced LVEF and ventricular dilation).
Intervention	Dietary supplements meeting predefined selection criteria. Interventions entering the main network meta-analysis were CoQ_10_ (30, 60, 90, and 150 mg/d), taurine, and n-3 PUFAs; the CoQ_10_ 60 mg/d + taurine combination was assessed only as exploratory pairwise evidence because its comparator was an active CoQ_10_ arm.
Comparator	Conventional treatment (CT), placebo, or no additional intervention.
Outcomes	LVEF, LVEDD, LVESD, NT-proBNP, NYHA class, and heart rate (HR).
Study Design	Randomized controlled trials (RCTs).

### Data extraction and risk-of-bias assessment

Two reviewers (Y.L. and R.L.) independently extracted data and assessed risk of bias, with disagreements resolved by a third reviewer (K.Y.). As noted above, all three are medical professionals in the cardiovascular field with more than five years of experience in evidence-based / systematic-review research. A predefined quality-assurance process was followed throughout: (i) a standardised data-extraction form and RoB 2 guidance notes were prepared in advance; (ii) before formal extraction, the reviewers completed focused training on the Cochrane Risk-of-Bias 2 (RoB 2) tool and piloted both the extraction form and RoB 2 judgements on a subset of included studies, with discrepancies discussed until consensus definitions were reached; (iii) dual independent review was then applied to all studies, and unresolved disagreements were adjudicated by the third reviewer; and (iv) a senior corresponding investigator supervised the process and spot-checked extracted data and domain judgements for completeness and consistency. Extracted items included first author, year, age, sex, study design, treatment and comparator definitions, dose, treatment duration, and final-timepoint outcome values. Where standard errors of the mean were reported, they were converted to standard deviations (SD). Risk of bias was assessed with the Cochrane Risk-of-Bias 2 (RoB 2) tool across its five domains (randomization process, deviations from intended interventions, missing outcome data, measurement of the outcome, selection of the reported result) [20]; judgments were recorded as low risk, some concerns, or high risk.

### Statistical analysis

NMA of the continuous outcomes (LVEF, LVEDD, LVESD, HR) was performed in Stata/MP 18.0 [21–23] using the network and mvmeta suite. A frequentist random-effects model was fitted by restricted maximum likelihood, assuming a single common heterogeneity variance across comparisons (exchangeable between-study covariance structure with common correlation ρ = 0.5); CT was the reference and the mean difference (MD) the effect measure. Global inconsistency was assessed by the design-by-treatment interaction test [24], and local inconsistency by node-splitting (side-splitting) [25]. Treatments were ranked by the surface under the cumulative ranking curve (SUCRA) [26] using Monte-Carlo simulation.

Multi-component regimens compared with an active single-component arm were analysed only as exploratory pairwise evidence and were not entered into the main NMA, because component-specific effects could not be isolated. Pre-specified subgroup factors were treatment duration, country, and CoQ_10_ dose; all subgroup and sensitivity analyses are exploratory/post-hoc and interpreted as hypothesis-generating. Sensitivity analyses examined robustness to (i) trials in which an active co-intervention was administered only to the intervention arm, and (ii) trials with implausibly small reported SDs for ventricular dimensions. Eligibility for these exclusions was determined from the extracted intervention, comparator, and variance data. Heterogeneity was quantified by I² and τ². For each treatment-versus-CT contrast, 95% prediction intervals (PIs) were calculated as $PI=MD\;\pm\;t_{k-2}\;\times\;\sqrt{\tau^{2}+\left(SE\right)^{2}}$, where MD is the pooled mean difference, SE is its standard error, k is the number of studies in the network, and t_(k−2)_ is the two-sided 95% critical value of the Student t distribution with k-2 degrees of freedom, to quantify the expected range of a future study’s true effect [27]. Certainty of evidence for key comparisons was rated with the Grading of Recommendations Assessment, Development and Evaluation (GRADE) approach [28]. A two-sided *P* < 0.05 was considered significant. Traditional meta-analyses and risk-of-bias graphics were produced in Review Manager 5.4 [29].

### Declaration of Artificial Intelligence (AI) use

In accordance with the journal’s policy regarding Artificial Intelligence Generated Content (AIGC), the authors declare that no generative artificial intelligence (AI) tools were used in developing any portion of this manuscript, including the formal research design, data analysis, or manuscript writing.

## Results

### Literature search

**Fig. 1:**
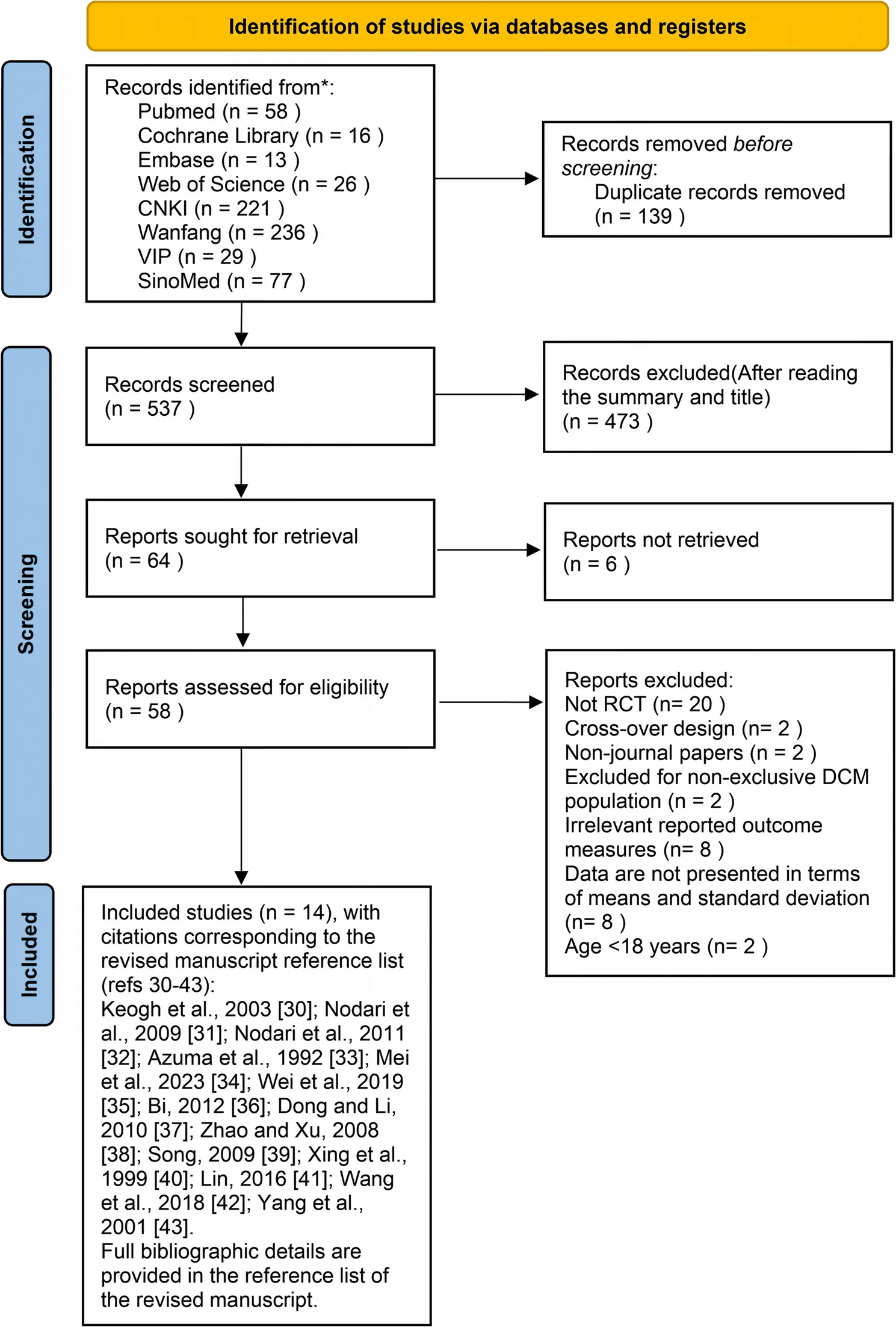
Document screening process and results. Note: The diagram follows the official PRISMA template; the 14 included RCTs are listed with citations (refs 30–43). Database-specific record counts, exclusion reasons at the full-text stage, and the 14 included RCTs with citations (refs 30–43) are shown in the figure / figure note

The screening process is shown in Fig. 1. Of 676 records initially retrieved, 14 RCTs (1,259 randomised participants; 1,220 contributed evaluable outcome data) were eligible for quantitative synthesis [30–43]. Among the 14 RCTs, 4 were published in English [30–33] and 10 in Chinese [34–43]; publication years ranged from 1992 to 2023. Applying the intervention eligibility rule, CoQ_10_, taurine, and n-3 PUFAs were retained; L-carnitine, creatine, thiamine, vitamins C and D, and selenium were excluded because no eligible DCM RCTs were identified. Candidate supplements and reasons for inclusion/exclusion are listed in Supplementary Table S1.

### Study characteristics and quality

**Fig. 2:**
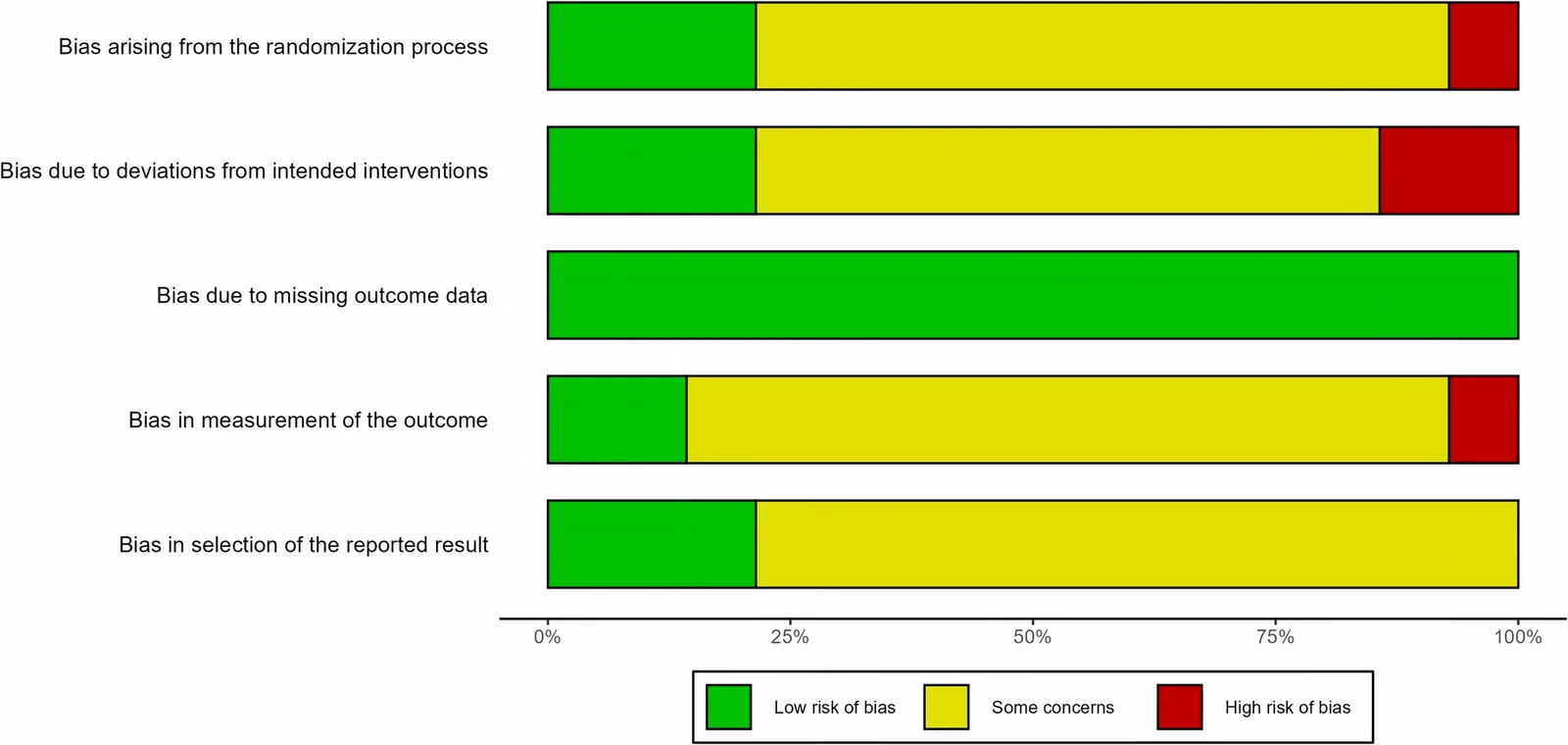
Risk of bias assessment included in the study

All included studies were RCTs comparing a dietary supplement (various CoQ_10_ doses, taurine, or n-3 PUFAs) with CT, placebo, or an active arm. Baseline characteristics are summarised in Supplementary Table S2. Risk of bias (RoB 2) is summarised in Fig. 2 (weighted summary bar chart) and Supplementary Figure S1 (traffic-light plot): about one-third of trials were double-blind and adequately concealed (low risk), whereas most Chinese open-label trials raised some concerns, particularly for the measurement of operator-dependent echocardiographic outcomes and the subjective NYHA assessment. Three trials had an active co-intervention administered only to the intervention arm: levosimendan with CoQ_10_ in Mei 2023 [34], Qiliqiangxin with CoQ_10_ in Song 2009 [39], and metoprolol with CoQ_10_ in Dong 2010 [37]; the corresponding control arms did not receive these agents. Dong 2010 administered CoQ_10_ 10–20 mg three times daily (30–60 mg/d); according to the dose-coding rule, this arm was represented as the CoQ_10_ 60 mg node in the network. The multi-component CoQ_10_ 60 mg + taurine regimen used an active CoQ_10_ comparator and was therefore analysed only as exploratory pairwise evidence.

### Network meta-analysis

#### LVEF

**Fig. 3:**
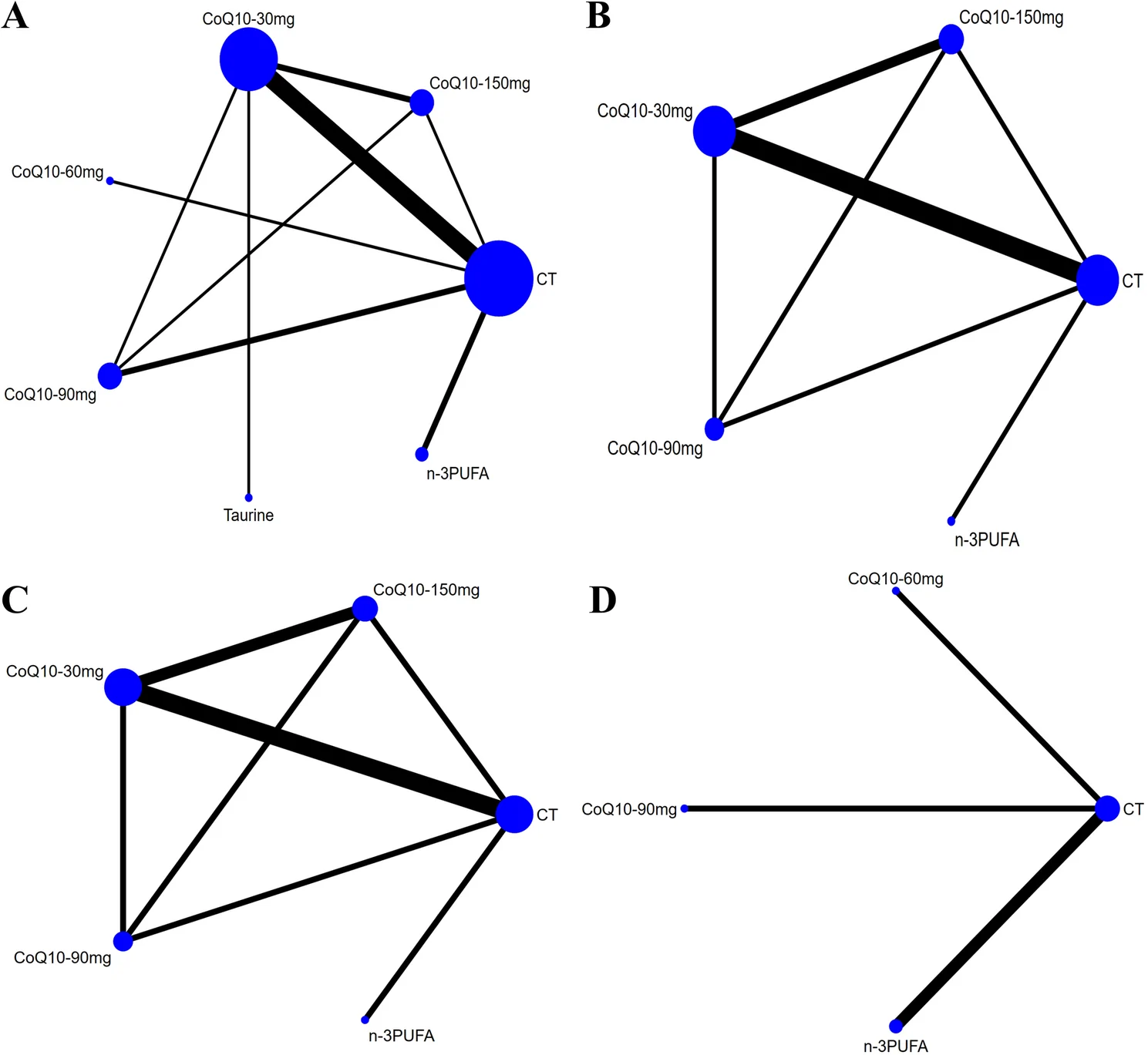
The evidence network of all papers on different treatments. Note: (**A**) LVEF, (**B**) LVEDD, (**C**) LVESD, (**D**) Heart rate. Line thickness reflects the number of studies, while node size represents the total sample size for each treatment

**Fig. 4:**
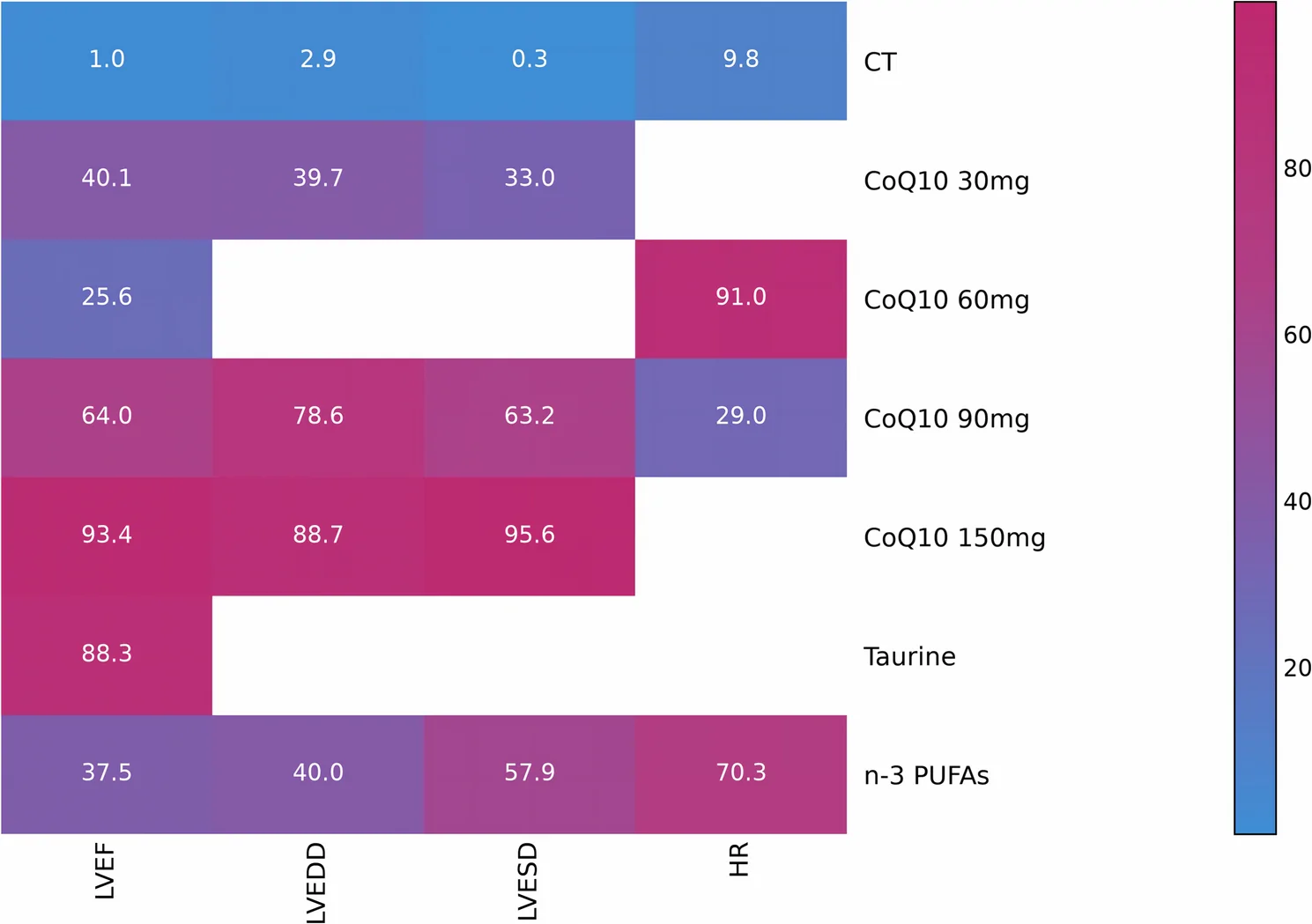
Heat map of SUCRA values for various intervention measures

**Fig. 5:**
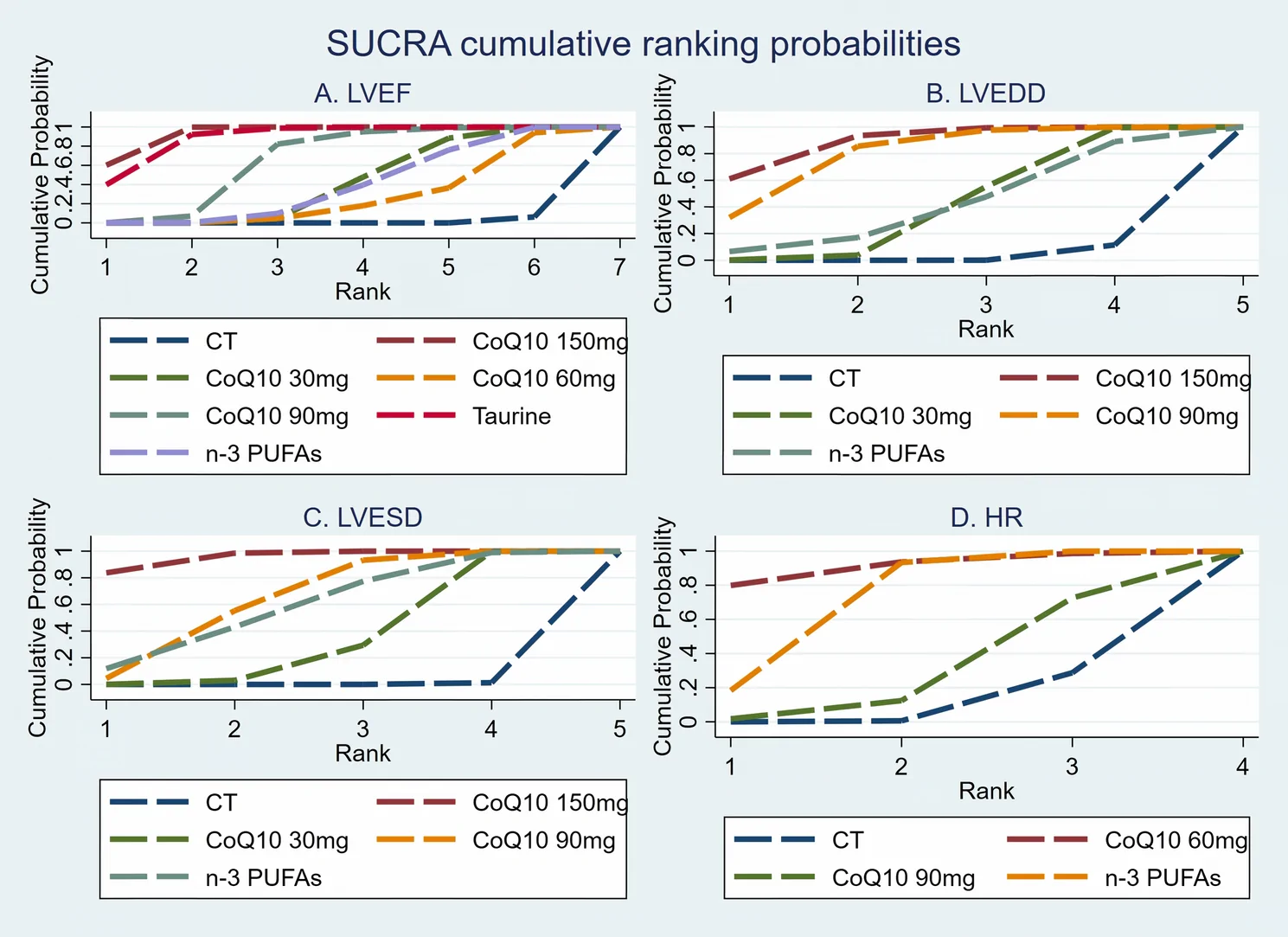
The SUCRA values for primary outcomes. Note: (**A**) LVEF, (**B**) LVEDD, (**C**) LVESD, (**D**) Heart rate

Twelve RCTs [31–42] informed the LVEF network of seven nodes (CT, CoQ_10_ 30, 60, 90 and 150 mg, taurine, and n-3 PUFAs; Fig. 3A). Compared with CT, significant improvements were seen for CoQ_10_ 150 mg (MD = 11.26, 95% confidence interval (CI) 8.55 ~ 13.98), taurine (10.50, 5.88 ~ 15.13), CoQ_10_ 90 mg (6.69, 4.00 ~ 9.38), CoQ_10_ 30 mg (4.50, 2.95 ~ 6.06) and n-3 PUFAs (4.21, 1.27 ~ 7.15); CoQ_10_ 60 mg was not significant (3.00, − 0.75 ~ 6.75). Head-to-head, CoQ_10_ 150 mg was superior to CoQ_10_ 30 mg (6.76, 3.95 ~ 9.57), CoQ_10_ 60 mg (8.26, 3.63 ~ 12.89), CoQ_10_ 90 mg (4.58, 1.39 ~ 7.77) and n-3 PUFAs (7.05, 3.01 ~ 11.09). Global inconsistency was borderline (design-by-treatment χ²(3) = 7.24, *P* = 0.065); node-splitting revealed local inconsistency in the CoQ_10_ 30 vs. 90 mg comparison (*P* = 0.042). By SUCRA, CoQ_10_ 150 mg ranked first (93.4%), followed by taurine (88.3%) and CoQ10 90 mg (64.0%). The high rank of taurine rests on a single 1992 trial [33] (*n* = 17) whose comparator was an active CoQ_10_ 30 mg arm rather than CT, so it is connected to CT only indirectly and should be interpreted with caution. Study-level forest plots for all NMA outcomes are provided in Supplementary Figures S2–S5. Full league tables of all pairwise comparisons are provided in Supplementary Tables S3-S6. SUCRA-based treatment rankings across outcomes are summarised in Fig. 4; Table 2, with corresponding SUCRA curves in Fig. 5. Corresponding 95% prediction intervals were considerably wider and, for the lower-ranked nodes, included no effect (for LVEF, CoQ_10_ 150 mg vs. CT 6.99 to 15.54, taurine 4.47 to 16.54, n-3 PUFAs − 0.25 to 8.67).

**Table 2:** The SUCRA values of each treatment modality

Treatment	LVEF	Rank	LVEDD	Rank	LVESD	Rank	HR	Rank
CoQ_10_ 150 mg	93.4	1	88.7	1	95.6	1	-	-
CoQ_10_ 90 mg	64.0	3	78.6	2	63.2	2	29.0	3
CoQ10 60 mg	25.6	6	-	-	-	-	91.0	1
CoQ_10_ 30 mg	40.1	4	39.7	4	33.0	4	-	-
Taurine	88.3	2	-	-	-	-	-	-
*n*−3 PUFAs	37.5	5	40.0	3	57.9	3	70.3	2
CT	1.0	7	2.9	5	0.3	5	9.8	4

#### LVEDD

Six RCTs [32, 34, 35, 39, 41, 42] informed the LVEDD network (Fig. 3B). Compared with CT, CoQ_10_ 150 mg (− 5.40, − 8.14 ~ − 2.66), CoQ_10_ 90 mg (− 4.79, − 7.59 ~ − 1.99) and CoQ_10_ 30 mg (− 2.42, − 4.22 ~ − 0.62) significantly reduced LVEDD; n-3 PUFAs did not (− 2.30, − 5.92 ~ 1.32). Global inconsistency was not significant (χ²(2) = 1.64, *P* = 0.44). SUCRA ranked CoQ_10_ 150 mg first (88.7%) and CoQ_10_ 90 mg second (78.6%).

#### LVESD

Five RCTs [32, 35, 39, 41, 42] informed the LVESD network (Fig. 3C). Compared with CT, CoQ_10_ 150 mg (− 6.31, − 8.60 ~ − 4.01), CoQ_10_ 90 mg (− 4.19, − 6.57 ~ − 1.81), n-3 PUFAs (− 3.90, − 7.15 ~ − 0.65) and CoQ_10_ 30 mg (− 2.51, − 4.19 ~ − 0.83) significantly reduced LVESD. However, global inconsistency was significant (χ²(2) = 9.58, *P* = 0.008), so the LVESD network estimates are downgraded and should be interpreted cautiously. SUCRA ranked CoQ_10_ 150 mg first (95.6%). As with LVEDD, the LVESD estimates were fragile to exclusion of trials with implausibly small reported SDs (see Sensitivity analyses).

#### Heart rate

Four RCTs [31, 32, 37, 40] informed a star-shaped HR network with no closed loops (Fig. 3D). Compared with CT, n-3 PUFAs significantly reduced HR (− 3.94, − 5.11 ~ − 2.77) and CoQ_10_ 60 mg also reduced HR (− 7.00, − 13.42 ~ − 0.58); CoQ_10_ 90 mg was not significant (− 1.17, − 4.90 ~ 2.56). By SUCRA, CoQ_10_ 60 mg ranked first (91.0%) but rested on a single trial with concomitant beta-blockade 37, whereas the n-3 PUFA estimate (SUCRA 70.3%) derived from two double-blind RCTs 31,32 and is the most robust.

### Pairwise meta-analysis of NT-proBNP and NYHA

#### NT-proBNP

**Fig. 6:**
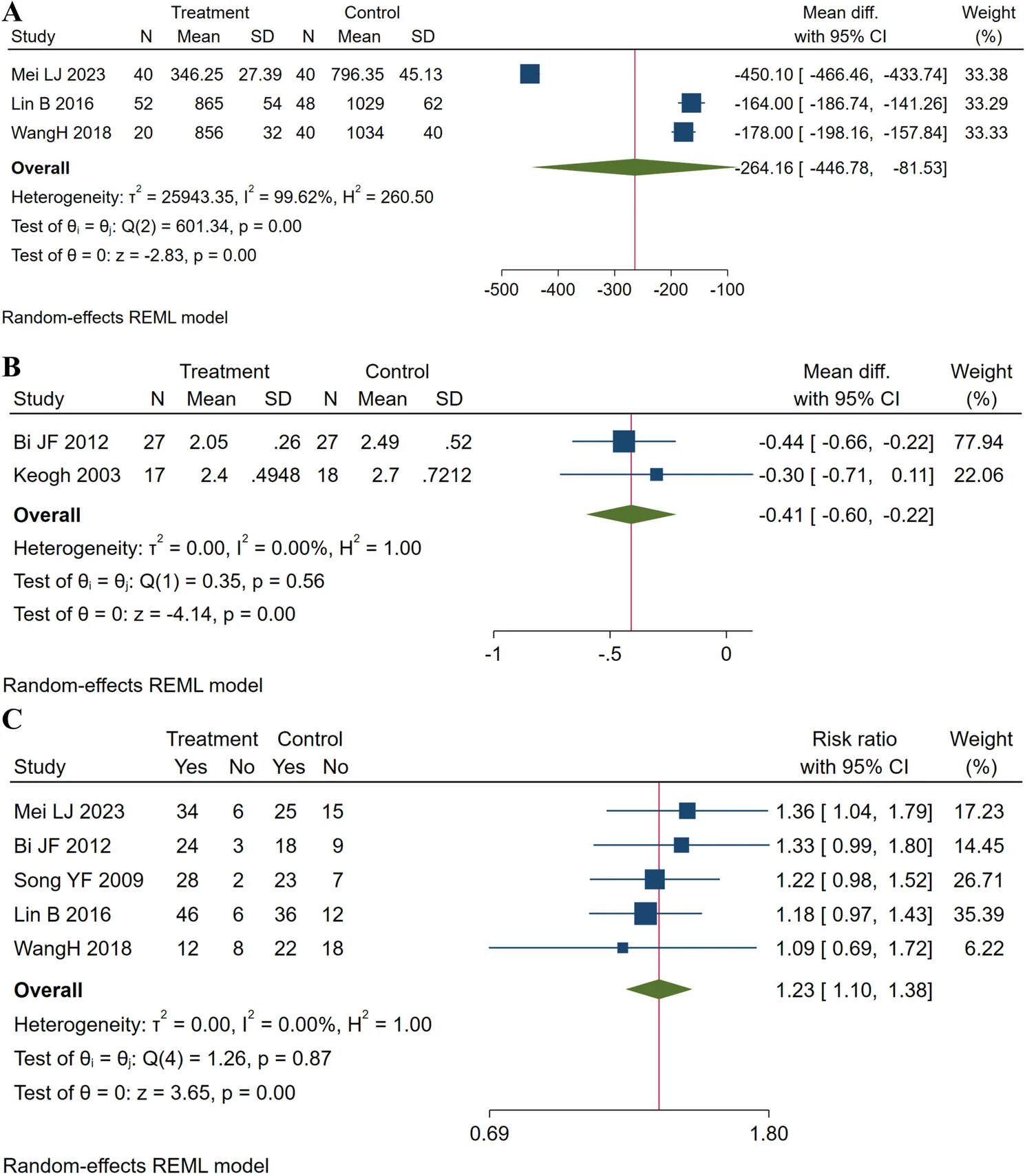
Random-effects REML pairwise meta-analyses of CoQ_10_ versus CT. Note: (**A**) the reduction in NT-proBNP in pg/mL with extreme heterogeneity driven by the levosimendan co-treated trial [34]; (**B**) the reduction in mean NYHA functional class; and (**C**) the risk ratio for improving by at least one NYHA class

Because the NT-proBNP network showed significant loop inconsistency and rested on only three recent Chinese trials [34, 41, 42], it was analysed by pairwise meta-analysis (CoQ_10_ vs. CT). The pooled effect was a reduction of − 264 pg/mL (95% CI − 447 ~ − 82) but with extreme heterogeneity (I² = 99.6%). This was driven by one trial co-administering levosimendan [34]; after excluding it, the effect was smaller and homogeneous (− 172 pg/mL, − 187 ~ − 157; I² = 0%). NT-proBNP results should therefore be interpreted as low-certainty and confounding-sensitive (Fig. 6A; Supplementary Table S7).

#### NYHA functional class

As NYHA is ordinal, it was synthesised by pairwise meta-analysis. CoQ_10_ versus CT reduced mean NYHA class by − 0.41 (95% CI − 0.60 ~ − 0.22; I² = 0%, two trials [30, 36]; Fig. 6B). In addition, the proportion of patients improving by ≥ 1 NYHA class favoured CoQ_10_ (RR 1.23, 1.10 ~ 1.38; I² = 0%, five trials [34, 36, 39, 41, 42]; Fig. 6C). The exploratory CoQ_10_ 60 mg + taurine combination [43] (single trial, active CoQ_10_ comparator) was associated with a further mean reduction of − 0.43 class and an improvement RR of 1.23 versus CoQ_10_ 60 mg, but the taurine-specific contribution cannot be isolated (Supplementary Table S7).

### Subgroup analyses

Exploratory, post-hoc subgroup pairwise meta-analyses of supplement-versus-CT effects on LVEF are summarised in Table 3. Only trials with a genuine CT or placebo comparator contributed; the taurine and combination trials could not form a versus-CT subgroup because their comparators were active CoQ_10_ arms. This left 10 RCTs contributing 12 supplement-versus-CT contrasts, with an overall effect of 5.36 (95% CI 3.73 ~ 6.99; I² = 74.8%). Of the three factors examined, only CoQ_10_ dose showed a statistically significant between-subgroup difference (interaction *P* < 0.001), consistent with a dose-response gradient in which the pooled effect rose from 4.32 (95% CI 3.35 ~ 5.28) for 30 mg to 12.00 (9.22 ~ 14.78) for 150 mg. Differences across treatment duration (interaction *P* = 0.329) and country (interaction *P* = 0.296) were not significant. Intervention type (CoQ_10_ vs. n-3 PUFAs) was collinear with country (the only n-3 PUFA trials were the two Italian studies [31, 32]), so it is not reported as a separate factor. Because these analyses were post-hoc and several strata rest on only one or two small trials, the subgroup findings are hypothesis-generating and should be interpreted with caution. Subgroup results for all outcomes, including LVEDD and LVESD, are given in Supplementary Table S8, with forest plots in Supplementary Figures S6–S10.

**Table 3:** Subgroup analyses for LVEF

Type of subgroup	k (contrasts)	MD (95%CI)	*P* (effect)	I^2^	Het. *P* (Q)	Interaction *P*
Overall	12	5.36 (3.73, 6.99)	< 0.001	74.8%	< 0.001	—
Duration						0.329
short-term	4	5.59 (3.50, 7.68)	< 0.001	15.5%	0.326	
medium-term	5	6.04 (2.64, 9.44)	< 0.001	89.4%	< 0.001	
long-term	3	4.00 (2.57, 5.43)	< 0.001	~ 0%	0.937	
Country						0.296
China	10	5.55 (3.65, 7.45)	< 0.001	78.2%	< 0.001	
Italy	2	4.11 (2.18, 6.03)	< 0.001	~ 0%	0.751	
CoQ_10_ dosage						< 0.001
CoQ_10_ 30 mg	6	4.32 (3.35, 5.28)	< 0.001	~ 0%	0.305	
CoQ_10_ 60 mg	1	3.00 (0.30, 5.70)	0.03	-	-	
CoQ_10_ 90 mg	2	5.92 (1.34, 10.51)	0.011	72.2%	0.058	
CoQ_10_ 150 mg	1	12.0 (9.22, 14.78)	< 0.001	-	-	

Values are mean differences (supplement − CT) in LVEF pooled by random-effects REML. K = number of supplement-versus-CT contrasts (not trials); the multi-arm trial WangH 2018 contributes three, its shared control being split across arms. P (effect), test that the pooled effect = 0; Het. P (Q), Cochran’s Q homogeneity test; Interaction P, between-subgroup difference test (Q_b), reported once per factor. Duration: short < 3, medium 3–< 6, long ≥ 6 months. – = not estimable for single-trial subgroups. Trials without a genuine CT/placebo comparator were excluded, including active-comparator trials [33, 35, 43]. Analyses were post-hoc and several strata rest on one or two small trials; findings are hypothesis-generating

*CT* conventional treatment

### Sensitivity analyses

For LVEF, excluding all three potentially confounded CoQ_10_ trials—Mei 2023 [34] (levosimendan), Song 2009 [39] (Chinese patent medicine), and Dong 2010 [37] (metoprolol added only to the intervention arm)—removed the CoQ_10_ 60 mg node and left the CoQ_10_ 30 mg versus CT estimate essentially unchanged (MD 4.18, 95% CI 2.52 to 5.85; SUCRA 30.8%), supporting robustness of the latter comparison. For HR, excluding Dong 2010 likewise removed the CoQ_10_ 60 mg node; n-3 PUFAs then ranked first (MD − 3.94 bpm, 95% CI − 5.11 to − 2.77; SUCRA 96.0%), whereas CoQ_10_ 90 mg remained non-significant (MD − 1.17 bpm, 95% CI − 4.90 to 2.56). For LVEDD and LVESD, excluding trials with implausibly small reported SDs markedly weakened or abolished the CoQ_10_ effects (CoQ_10_ 30 mg versus CT became − 2.80 and non-significant), indicating that the ventricular-dimension findings are fragile and partly driven by questionable variance estimates. For NT-proBNP, excluding the levosimendan-confounded trial reduced the effect from − 264 to − 172 pg/mL and removed all heterogeneity (I² 0%). A formal era-restricted or placebo-only NMA was precluded because such restrictions fragment the network (taurine is informed solely by a 1992 trial, and several nodes lose their only connection to CT). The corresponding sensitivity-analysis networks are shown in Supplementary Figures S11–S14.

### Certainty of evidence (GRADE)

Certainty of evidence for the key comparisons is summarised in Supplementary Table S9. It was moderate for n-3 PUFAs (double-blind placebo-controlled trials), low for CoQ_10_ 30 mg vs. CT (LVEF) and for the NYHA improvement RR, and very low for most other comparisons—downgraded for risk of bias (open-label trials, suspect SDs), inconsistency (LVESD; local LVEF), indirectness (taurine/combination via active comparators), imprecision (single-trial nodes), and suspected reporting bias.

### Adverse reactions

Adverse events were collected from the included RCTs and were predominantly mild (dizziness, gastrointestinal discomfort, cold extremities, nausea, palpitations) and self-limiting (Table 4; detailed per-study, per-arm counts in Supplementary Table S10). Because the trials reported only raw counts without consistent denominators, follow-up duration, or severity grading, event rates and between-group comparisons cannot be reliably estimated; the figures should be read as crude counts over heterogeneous follow-up (2–12 weeks). An appraisal using the Consolidated Standards of Reporting Trials harms extension (CONSORT-Harms) [44] confirmed generally poor safety reporting.

**Table 4:** Adverse events of different treatments

Treatment	Dizziness	GI discomfort	Cold extremities	Nausea	Palpitations
CT	2	-	-	-	-
coQ_10_ 60 mg	-	2	1	-	-
coQ_10_ 90 mg	-	-	-	2	
coQ_10_ 150 mg	-	-	-	3	1

### Prediction intervals

The 95% prediction intervals were generally wider than the corresponding confidence intervals, and several included the null, reflecting sparse networks and between-study heterogeneity. Full prediction intervals for all NMA contrasts are reported in Supplementary Table S11.

### Small-study effects and publication bias

**Fig. 7:**
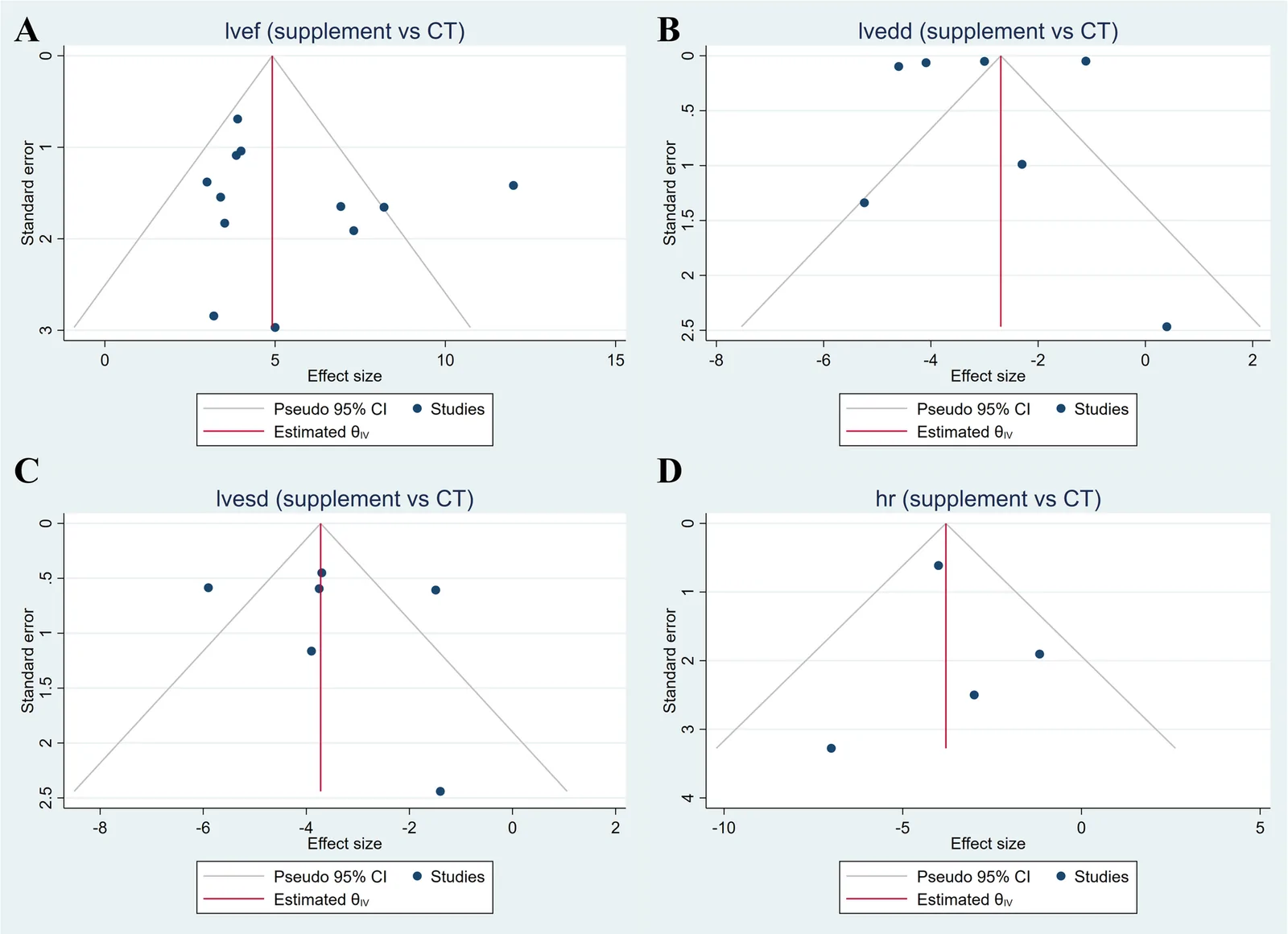
Funnel plot for publication bias in all outcomes. Note: (**A**) LVEF, (**B**) LVEDD, (**C**) LVESD, (**D**) Heart rate

Funnel plots of supplement-versus-CT contrasts are shown in Fig. 7. For LVEF, the only outcome with enough trials for meaningful assessment, the plot was approximately symmetrical and Egger’s test was non-significant (*P* = 0.81). For the remaining outcomes the small number of trials (k ≤ 7) precludes reliable assessment, and apparent asymmetry largely reflects clinical heterogeneity and selective outcome reporting rather than classical publication bias.

## Discussion

### Summary of main results

This study is, to our knowledge, the first network meta-analysis of dietary supplements specifically in adults with DCM, synthesising 14 RCTs. Across the ventricular-function outcomes, CoQ_10_ 150 mg/d was consistently top-ranked, while taurine also ranked highly for LVEF. However, firm dose-response inference was precluded by the sparse and heterogeneous evidence supporting individual dose nodes. For heart rate, n-3 PUFAs provided the most robust reduction, supported by two double-blind RCTs and ranking first in the co-intervention sensitivity analysis (SUCRA 96.0%). In pairwise analyses, CoQ_10_ lowered NT-proBNP and improved NYHA functional status, although the NT-proBNP effect was sensitive to a co-intervention-confounded trial. Overall, these findings should be regarded as hypothesis-generating rather than as identifying a single optimal regimen.

### Comparison with previous evidence

Overall, our findings are broadly consistent with prior synthesised evidence that nutritional interventions, including CoQ_10_ and n-3 PUFAs, can favourably affect cardiac function and related biomarkers in heart-failure populations [15, 45, 46]. However, earlier reviews rarely focused on idiopathic/non-ischaemic DCM alone and seldom resolved CoQ_10_ by dose within a formal network, so comparative ranking and dose–response relationships remained uncertain. Using NMA in a DCM-restricted population, we provide dose-resolved estimates and simultaneous ranking across CoQ_10_ doses, taurine and n-3 PUFAs for multiple structural and functional endpoints.

A notable feature is that the LVEF gains for higher-dose CoQ_10_ in this network appear larger than pooled effects reported in several mixed heart-failure CoQ_10_ meta-analyses, which often estimate more modest average improvements. Several factors may explain this discrepancy and warrant caution rather than direct extrapolation. One factor is that, most included CoQ_10_ trials were small, open-label Chinese studies, a setting in which treatment-effect inflation is well recognised. Another consideration is that the common CT comparator aggregates placebo, no add-on therapy and historically heterogeneous “conventional therapy,” so absolute effects versus contemporary guideline-directed medical therapy may be smaller than network estimates versus weaker backgrounds suggest. A mechanistic possibility is that DCM myocardium is characterised by particularly pronounced mitochondrial and bioenergetic impairment relative to some other heart-failure phenotypes, which could theoretically increase responsiveness to metabolic adjuncts, but this mechanistic plausibility does not substitute for larger placebo-controlled confirmation. Finally, it should be noted that classic Western CoQ_10_ trials in chronic heart failure have not uniformly demonstrated clinically meaningful systolic improvement, underscoring that our favourable CoQ_10_ ranks should be interpreted against a mixed external evidence base rather than as settled superiority. Taken together, the dose–response signal for CoQ_10_ in DCM is clinically intriguing but remains low-to-very-low certainty for most high-ranking comparisons.

For n-3 PUFAs, the moderate LVEF benefit and clearer heart-rate reduction align with prior DCM/heart-failure trials and meta-analytic evidence supporting electrophysiological stabilisation and remodeling effects of omega-3 fatty acids [32, 46]. In our GRADE appraisal, n-3 PUFAs uniquely reached moderate certainty for key comparisons because the contributing evidence included double-blind, placebo-controlled DCM trials, contrasting with the predominantly open-label CoQ_10_ evidence base.

### Pathophysiological explanation of mechanisms

The core pathophysiology of DCM involves mitochondrial dysfunction, oxidative stress and disordered myocardial energetics. The supplements evaluated here converge on these pathways through distinct but complementary mechanisms. CoQ_10_ is an essential electron carrier in the mitochondrial respiratory chain, shuttling electrons from complexes I/II to III and supporting the proton motive force required for ATP synthesis [47, 48] ; its reduced form is also a potent lipophilic antioxidant that limits ROS-mediated lipid peroxidation and protects mitochondrial membranes [49]. The observed CoQ_10_ dose–response for LVEF is consistent with dose-dependent replenishment of myocardial CoQ_10_ stores and correction of bioenergetic deficits, although pharmacokinetics, formulation bioavailability and background therapy remain important modifiers.

Taurine, a conditionally essential amino acid enriched in myocardium, contributes to calcium handling, osmoregulation and anti-inflammatory homeostasis [50]. Combined CoQ_10_ –taurine strategies are biologically attractive, CoQ_10_ supporting mitochondrial energetics while taurine stabilises excitation–contraction coupling [51, 52], yet in the present evidence the taurine and combination nodes largely depended on active CoQ_10_ comparators rather than CT/placebo, so taurine-specific incremental benefit cannot be isolated and high SUCRA ranks for these nodes should not be over-interpreted.

n-3 PUFAs incorporate into membrane phospholipids, favour production of specialised pro-resolving mediators, and modulate cardiac ion-channel behavior [53, 54], which may collectively reduce arrhythmogenic susceptibility and support reverse remodeling. These mechanisms are compatible with the relatively robust heart-rate benefit of n-3 PUFAs in our network and with prior DCM trials reporting electrophysiological and functional gains.

### Subgroup findings and heterogeneity

Exploratory post-hoc subgroup analyses of supplement-versus-CT contrasts for LVEF suggested that CoQ_10_ dose was the only factor with a statistically significant between-subgroup difference (interaction *P* < 0.001), with pooled effects rising from approximately 4% at 30 mg/d to about 12% at 150 mg/d. Differences by treatment duration and country were not significant, although Chinese open-label trials contributed most of the heterogeneity and Italian n-3 PUFA trials were more homogeneous. Because several strata rest on one or two small trials and analyses were not multiplicity-controlled, these patterns are hypothesis-generating. Clinical implications remain tentative: short-term LVEF gains may partly capture reversible contractile reserve, whereas durable reverse remodeling (LVEDD/LVESD) likely requires longer exposure, yet our ventricular-dimension estimates were fragile to exclusion of trials with implausibly small reported standard deviations and therefore cannot firmly define an optimal treatment window.

### Safety assessment and reporting quality

Across included RCTs, reported adverse events were mainly mild (dizziness, gastrointestinal discomfort, cold extremities, nausea, palpitations) and self-limiting, and no clear dose-related excess of harm was evident for CoQ_10_ 150 mg/d within the short follow-up available. However, safety reporting quality was generally poor when appraised against CONSORT-Harms principles: studies typically provided crude counts without consistent denominators, follow-up time, standardised definitions, severity grading or attribution, precluding reliable rate estimation or comparative harm assessment. Future DCM supplement trials should adopt CONSORT-Harms 2022 standards [55] to improve the completeness and interpretability of safety data.

### Limitations

Comparator exchangeability is the central threat to inference. Collapsing placebo, no add-on therapy and era-varying “conventional treatment” into a single CT node across 1992–2023 means that secular improvements in guideline-directed therapy (beta-blockers, ACE inhibitors/ARBs/ARNIs, mineralocorticoid antagonists, SGLT2 inhibitors) can alter outcomes independently of the supplement under study. Because virtually all network contrasts are anchored on CT, this limitation is structural rather than peripheral; era- or placebo-restricted NMAs fragment key nodes (for example, taurine rests on a single 1992 active-comparator trial [33]) and therefore could not fully resolve the problem analytically.

Several influential nodes rest on single small trials, and taurine/combination comparisons were often linked through active CoQ_10_ rather than CT, inflating indirect ranks. Network inconsistency was significant for LVESD (global *P* = 0.008) and local for LVEF (CoQ_10_ 30 vs. 90 mg, *P* = 0.042). Ventricular-dimension findings were sensitive to questionable variance reporting. Language/geographic concentration (10/14 RCTs Chinese; many open-label and non-PubMed-indexed) raises concerns about reporting and small-study bias. Follow-up was short (commonly 4–12 weeks; one trial [39], only two weeks, arguably too short to expect a true change in LVEF or remodeling), hard endpoints (death, hospitalisation) were absent, and safety ascertainment was incomplete. Strict eligibility and limited genetic stratification further constrain generalisability. Finally, GRADE certainty was moderate only for selected n-3 PUFA comparisons and low or very low for most CoQ_10_ dose comparisons.

### Implications for future research

Priority should be given to large, international, placebo-controlled RCTs of CoQ_10_ (including head-to-head dose comparisons that test the 90–150 mg/d range) and n-3 PUFAs in well-characterised DCM, conducted against standardised contemporary background therapy. Trials should use longer follow-up, pre-specify hard clinical endpoints, analyse NYHA with ordinal-appropriate methods, report safety according to CONSORT-Harms [44, 55], and ideally incorporate mechanistic biomarkers of myocardial energetics and oxidative stress. Until such evidence accrues, SUCRA rankings from the present network should inform research prioritisation rather than routine dose selection.

## Conclusion

This network meta-analysis suggests that dietary supplementation may provide meaningful adjunctive benefit in DCM. In this network, CoQ_10_ 150 mg/d showed the most favourable overall profile for systolic function and ventricular dimensions, while n-3 PUFAs offered the most reliable benefit for heart rate; a temporal separation between early functional improvement and later structural reversal was suggested but not statistically confirmed. Given small samples, predominantly Chinese open-label trials, a heterogeneous and time-varying comparator, local inconsistency, and incomplete safety reporting, these results are hypothesis-generating and require confirmation in larger, well-designed RCTs with longer follow-up.

## Supplementary Information

Supplementary Material 1.

## Data Availability

All data analysed in this study were extracted from previously published randomized controlled trials, which are cited in the reference list and were accessed through publicly available databases (PubMed, the Cochrane Library, Embase, Web of Science, CNKI, Wanfang, VIP, and SinoMed). The aggregated data supporting the findings of this study are available within the article and its Supplementary Information files. Candidate supplement selection is summarised in Supplementary Table S1, and baseline characteristics of the included studies are provided in Supplementary Table S2. Network meta-analysis league tables and pairwise comparison results are presented in Supplementary Tables S3–S7; subgroup analyses, GRADE assessments, adverse-event data, and 95% prediction intervals are provided in Supplementary Tables S8–S11. The full search strategy is provided in Appendix S1. The review protocol was registered with PROSPERO (CRD420261351179). Additional data and the statistical analysis files (Stata/Review Manager) are available from the corresponding author on reasonable request.

## Abbreviations

DCM: Dilated cardiomyopathy
RCT: Randomized controlled trial
NMA: Network meta-analysis
LVEF: Left ventricular ejection fraction
LVEDD: Left ventricular end-diastolic diameter
LVESD: Left ventricular end-systolic diameter
NT-proBNP: N-terminal pro-B-type natriuretic peptide
NYHA: New York Heart Association class
HR: Heart rate
CoQ_10_: Coenzyme Q_10_
n-3 PUFAs: n-3 polyunsaturated fatty acids
CT: Conventional treatment
PICOS: Population/Intervention/Comparator/Outcomes/Study design
PRISMA: Preferred Reporting Items for Systematic Reviews and Meta-Analyses
PROSPERO: International prospective register of systematic reviews
SUCRA: Surface under the cumulative ranking curve
MD: Mean difference
RR: Risk ratio
CI: Confidence interval
RoB 2: Cochrane Risk-of-Bias 2
GRADE: Grading of Recommendations Assessment, Development and Evaluation
ATP: Adenosine triphosphate
ROS: Reactive oxygen species
AE: Adverse event
