# Supplementary material for: Comparative effectiveness of dietary supplements in dilated cardiomyopathy: a systematic review and network meta-analysis
Source: BMC Cardiovasc Disord. 2026 Aug 27;26:907. doi: 10.1186/s12872-026-06509-x (PMC13656484; doi:10.1186/s12872-026-06509-x)
Supplement: Supplementary file 1 [file 12872_2026_6509_MOESM1_ESM.docx]

**Supplementary Materials**

**Contents**

**Supplementary Table S1.** Candidate dietary supplements and reasons for inclusion/exclusion

**Supplementary Table S2.** Baseline characteristics of included studies

**Supplementary Tables S3–S6.** League tables of NMA (LVEF, LVEDD, LVESD, HR)

**Supplementary Table S7.** Pairwise meta-analysis of NT-proBNP and NYHA

**Supplementary Table S8.** Subgroup analyses (LVEF / LVEDD / LVESD)

**Supplementary Table S9.** GRADE certainty-of-evidence summary

**Supplementary Table S10.** Adverse events by study and arm (detailed)

**Supplementary Table S11.** 95% Prediction intervals for the network meta-analyses (vs CT)

**Supplementary Figure S1.** Risk of bias (RoB 2) traffic-light plot

**Supplementary Figures S2–S5.** NMA forest plots (LVEF/LVEDD/LVESD/HR)

**Supplementary Figures S6–S10.** Subgroup forest plots (post-hoc, supplement vs CT)

**Supplementary Figure S11.** LVEF network excluding all three confounded CoQ_10_ trials.

**Supplementary Figures S12–S13.** LVEDD/LVESD network excluding small-SD trials.

**Supplementary Figure S14.** HR network after exclusion of a trial with an intervention-only co-treatment.

**Appendix S1.** Search strategy

## Supplementary Table S1. Candidate dietary supplements and reasons for inclusion/exclusion

| **Supplement** | **Mechanistic rationale** | **RCT evidence in DCM** | **Inclusion decision and rationale** |
| --- | --- | --- | --- |
| **Coenzyme Q_10_ (CoQ_10_)** | Mitochondrial electron carrier; lipophilic antioxidant; improves ATP synthesis | Yes  (multiple) | Included: most studied supplement in DCM; dose-stratified (30/60/90/150 mg/d) |
| **Taurine** | Calcium homeostasis; osmoregulation; anti-inflammatory; antifibrotic | Yes  (1: Azuma 1992) | Included: single RCT; very low certainty (n=17; comparator CoQ_10_ 30 mg, not CT) |
| **n-3 PUFAs (omega-3)** | Membrane stabilisation; anti-inflammatory; antiarrhythmic; modulates autonomic tone | Yes  (2: Nodari 2009, 2011) | Included: two double-blind placebo-controlled RCTs; highest-certainty evidence |
| **Multi-component combination**  **(CoQ_10_ + taurine + herbs)** | Synergistic metabolic + calcium + anti-inflammatory effects | Yes  (1: Yang YZ 2001) | Included as exploratory only: multi-component; taurine effect inseparable; active comparator, not CT/placebo |
| **L-Carnitine** | Fatty acid transport into mitochondria; enhances β-oxidation | No RCT in DCM | Excluded: no RCT reporting cardiac function/structure outcomes in adult DCM |
| **Vitamin C (ascorbic acid)** | Water-soluble antioxidant; ROS scavenging | No RCT in DCM | Excluded: no RCT reporting cardiac outcomes specifically in DCM (studies in broader HF ineligible per PICOS) |
| **Vitamin D** | Calcium regulation; RAAS suppression; anti-inflammatory | No RCT in DCM | Excluded: no RCT reporting cardiac outcomes in DCM (observational/HF studies do not meet inclusion criteria) |
| **Nitrates** | NO donor; vasodilation; preload reduction | No supplement-type RCT in DCM | Excluded: used as conventional therapy, not dietary supplement |
| **Selenium** | Glutathione peroxidase component; antioxidant | No RCT in DCM | Excluded: no RCT in DCM (Keshan disease is distinct cardiomyopathy aetiology) |
| **Thiamine (Vitamin B1)** | Cofactor for pyruvate dehydrogenase; myocardial energy metabolism | No RCT in DCM | Excluded: no RCT in DCM (HFrEF studies exist but aetiology differs) |
| **Creatine** | Phosphocreatine energy shuttle; ATP buffering | No RCT in DCM | Excluded: no RCT reporting cardiac outcomes in DCM |

**Note:** Candidate supplements identified via systematic search; retained if ≥1 RCT reported cardiac outcomes in adult DCM and a mechanistic rationale existed.

**Supplementary Table S2. Baseline characteristics of included studies**

| **ID** | **Paper** | **Diagnostic criteria** | **Sample size (Control group/Treatment group)** | **Gender**  **(M/F)** | **Control group**  **(M/F)** | **Treatment group**  **(M/F)** | **Intervention**  **Control group** | **Intervention**  **Treatment group** | **Dose** | **Age (years)**  **Control group** | **Baseline LVEF (%) Control group** | **Age (years)**  **Treatment group** | **Baseline LVEF (%) Treatment group** | **Duration of treatment (month）** | **Blinding / Design** | **Country** | **Background therapy** |
| --- | --- | --- | --- | --- | --- | --- | --- | --- | --- | --- | --- | --- | --- | --- | --- | --- | --- |
| 1 | Keogh 2003 | Symptomatic heart failure of NYHA class II or III due to ischemic, valvular, or idiopathic dilated cardiomyopathy, with impaired left ventricular systolic function (LVEF < 40%) | 18/17/- | 27/8 | 15/3 | 12/5 | Placebo | CoQ_10_ 50mg tid | 150 mg/d (tid) | 61±9 | Not reported (FS only) | 62±7 | Not reported (FS only) | 3 | Double-blind, placebo-controlled | Australia | ACE-I 100%; no β-blockers; amiodarone, nitrates, diuretics, warfarin, hydralazine, digoxin |
| 2 | Nodari 2009 | Patients with IDC, LVEF ≤45%, and frequent or repetitive ventricular arrhythmias on optimal medical therapy | 22/22/- | 40/4 | 19/3 | 21/1 | Olive oil placebo | n-3 PUFA (EPA+DHA 850–882 mg/d) | 850–882 mg/d (5 caps/d ×1mo, then 1 cap/d) | 64.82±9.46 | 35.8±9.7 | 61.09±11.22 | 36.5 ± 8.6 | 6 | Double-blind, placebo-controlled | Italy | β-blockers 100%; ACE-I 73–77%; ARB 23–27%; furosemide 91–95%; spironolactone 50–54%; amiodarone 200mg/d 91–95% |
| 3 | Nodari 2011 | Patients with NICM, LVEF ≤45%, NYHA class I–II | 66/67/- | 120/13 | 56/10 | 64/3 | Olive oil  placebo | n-3 polyunsaturated fatty acids  (Omacor) | 850–882 mg/d (loading 5 caps×1mo, then 2 caps/d) | 64±9 | 37±6 | 61±11 | 36±7 | 12 | Double-blind, placebo-controlled | Italy | ACE-I/ARB 100%; β-blockers 100% (carvedilol ~42mg, bisoprolol ~8–9mg); furosemide 100%; spironolactone 57–64%; amiodarone 40–53%; ICD 40%; CRT 25–33% |
| 4 | Azuma J 1992 | Chronic congestive heart failure, LVEF<50% (etiologies: ischemic or IDC) | 7/10/- | 11/6 | - | - | CoQ_10_ 30 mg/d (10mg tid) | Taurine 3 g/d | 3,000 (1g tid) | - | 39±3 | - | 39±3 | 2 | Double-blind RCT | Japan | Digoxin, diuretics, vasodilators; no ACE-I/β-blockers (1992 standard) |
| 5 | Mei LJ 2023 | NYHA functional class III–IV, LVEF <40% | 40/40/- | 48/32 | 22/18 | 26/14 | Conventional treatment (CT) | CoQ_10_ 30 mg/d + levosimendan 12.5mg IV (single dose) | 30 (tid) | 66.02±5.49 | 36.34±3.46 | 67.34±4.58 | 35.27±4.15 | 1 | Open-label RCT | China | Sacubitril/valsartan 50mg bid; metoprolol 47.5mg qd; digoxin 0.125mg qd; furosemide 20mg qd; spironolactone 20mg qd |
| 6 | Wei YJ 2019 | Exclusion of NYHA class IV heart failure, CAD, valvular heart disease, and other relevant conditions | 46/34/- | 50/30 | 28/18 | 22/12 | CoQ_10_ 30 mg/d | CoQ_10_ 150 mg/d | 150 (tid) | 48.8±15.8 | 38.6 ± 9.21 | 50.2±14.2 | 38.8 ± 9.43 | 6 | Open-label RCT | China | Diuretics, RAAS blockers, spironolactone, β-blockers; inotropes as needed |
| 7 | Bi JF 2012 | Moderate to severe heart failure (NYHA functional class II–IV) | 27/27/- | 40/14 | 21/6 | 19/8 | CT (digoxin, HCTZ, spironolactone, enalapril, metoprolol) | CoQ_10_ 30 mg/d | 30 (tid) | 50±12 | 26.33±10.43 | 49±13 | 25.77±12.59 | 2 | Open-label RCT | China | Digoxin, HCTZ, spironolactone, enalapril (ACE-I), metoprolol (β-blocker) |
| 8 | Dong ZB 2010 | NYHA functional class II–III, sinus rhythm, with exclusion of hypertension and coronary artery disease | 33/33/- | 42/24 | 21/12 | 21/12 | CT (digoxin, HCTZ, captopril) | CoQ_10_ 30–60 mg/d (10–20 mg tid) + metoprolol 6.25–50 mg/d | CoQ_10_ 30–60 mg/d (10–20 mg tid) | 52±8.3 | Not reported | 51±7.1 | Not reported | 3 | Open-label RCT | China | Treat: metoprolol 6.25–50 mg/d + CoQ_10_ 30–60 mg/d + digoxin + HCTZ + captopril / Ctrl: digoxin + HCTZ + captopril |
| 9 | Zhao HM 2008 | NYHA functional class II–IV with overt cardiomegaly | 60/62/- | 68/54 | 32/28 | 36/26 | Simvastatin 10mg/d | CoQ_10_ 30 mg/d + simvastatin 10mg/d | 30 (tid) | 58.5±11.4 | 37.18±7.31 | 60.5±10.2 | 37.86±6.25 | 6 | Open-label RCT | China | Diuretics, ACE-I, β-blockers, digitalis; BOTH arms received simvastatin 10mg/d |
| 10 | Song YF 2009 | DCM complicated with heart failure, NYHA functional class II–IV | 30/30/- | 32/28 | - | - | CT (digitalis, β-blocker, ACE-I, nitroprusside + furosemide + dopamine) | CoQ_10_ 30 mg/d + Qiliqiangxin 4 caps tid | 30 (tid) | - | 35.9 ± 9.5 | - | 34.2 ± 8.9 | 0.5 | Open-label RCT | China | Digitalis, metoprolol (β-blocker), enalapril/captopril (ACE-I), nitroprusside, furosemide, dopamine |
| 11 | Xing HY 1999 | Unclear | 24/24/- | 23/21 | - | - | CT (oxygen, Na restriction, digitalis, diuretics, vasodilators, antibiotics) | CoQ_10_ 90 mg/d | 90 (tid, 30mg×3) | - | 24.18±4.17 | - | 24.85±3.16 | 2 | Open-label RCT | China | Oxygen, Na restriction, digitalis, diuretics, vasodilators, antibiotics (if infected), electrolyte correction |
| 12 | Lin B2016 | LVEDD >55mm (male) or >50mm (female), LVEF ≤40% | 48/52/- | 58/42 | - | - | CT (ACE-I, metoprolol, digoxin, furosemide, HCTZ, spironolactone) | CoQ_10_ 30 mg/d | 30 (tid) | 42.4±4.7 | 34.8±3.1 | 43.1±5.2 | 35.2±3.5 | 3 | Open-label RCT | China | ACE-I, metoprolol (β-blocker), digoxin, furosemide, HCTZ, spironolactone |
| 13 | WangH 2018 | LVEDD >55mm (male) or >50mm (female) and LVEF ≤40% measured by transthoracic echocardiography | 40/20/20/20 | 55/45 | 20/20 | 35/25 | Conventional treatment (CT) | CoQ_10_ 30 mg/d, CoQ_10_ 90 mg/d, CoQ_10_ 150 mg/d | 30 / 90 / 150 (tid) | 64±15 | 29.6±3.9 | 65±5 | 29.8±4.0  29.6±3.3  29.0±3.7 | 3 | Open-label RCT | China | ACE-I/ARB; β-blockers; diuretics |
| 14 | Yang YZ 2001 | Unclear | 156/164 | 210/110 | 104/52 | 106/58 | CT + CoQ_10_ 60 mg/d | Combo: CoQ_10_ 60 mg/d + taurine + Astragalus + Shengmai injection | CoQ_10_ 60 (20mg tid) | 49.8±11.7 | 27.35±11.47 | 46.4±10.5 | 26.6±13.11 | 6 | Open-label RCT | China | ACE-I, β-blockers, diuretics, digitalis, vasodilators; BOTH arms received CoQ_10_ 60mg/d; TCM-WM arm added taurine + herbs |

**Note:** M/F, male/female; NYHA, New York Heart Association class; LVEF, left ventricular ejection fraction; FS, fractional shortening; LVEDD, left ventricular end-diastolic diameter; DCM, dilated cardiomyopathy; IDC, idiopathic dilated cardiomyopathy; NICM, non-ischemic cardiomyopathy; CAD, coronary artery disease; CoQ_10_, coenzyme Q_10_; n-3 PUFA(s), n-3 polyunsaturated fatty acids; EPA, eicosapentaenoic acid; DHA, docosahexaenoic acid; CT, conventional treatment; ACE-I, angiotensin-converting enzyme inhibitor; ARB, angiotensin receptor blocker; RAAS, renin-angiotensin-aldosterone system; HCTZ, hydrochlorothiazide; ICD, implantable cardioverter-defibrillator; CRT, cardiac resynchronization therapy; IV, intravenous; tid, three times daily; bid, twice daily; qd, once daily; mg/d, milligrams per day; TCM-WM, traditional Chinese medicine-Western medicine. Sample-size and Gender columns reflect randomised participants; where trials reported loss to follow-up (Nodari 2009, n=3; Yang YZ 2001, n=36), fewer participants contributed evaluable outcome data (total randomised, 1,259; total evaluable, 1,220). Dong 2010 reported CoQ_10_ 10–20 mg tid (30–60 mg/d) and metoprolol starting at 6.25 mg/d, titrated to a maximum of 25 mg bid (50 mg/d).

**Supplementary Table S3 Results of network Meta-Analysis(LVEF)**

| **CoQ_10_ 150mg** |  |  |  |  |  |  |
| --- | --- | --- | --- | --- | --- | --- |
| 0.76 (-4.43, 5.95) | **Taurine** |  |  |  |  |  |
| 4.58 (1.39, 7.77) | 3.82 (-1.42, 9.06) | **CoQ_10_ 90mg** |  |  |  |  |
| 7.05 (3.01, 11.09) | 6.29 (0.82, 11.76) | 2.47 (-1.53, 6.48) | **n-3 PUFAs** |  |  |  |
| 6.76 (3.95, 9.57) | 6.00 (1.64, 10.36) | 2.18 (-0.73, 5.09) | -0.29 (-3.60, 3.01) | **CoQ_10_ 30mg** |  |  |
| 8.26 (3.63, 12.89) | 7.50 (1.55, 13.46) | 3.69 (-0.93, 8.30) | 1.21 (-3.56, 5.98) | 1.50 (-2.56, 5.57) | **CoQ_10_ 60mg** |  |
| 11.26 (8.55, 13.98) | 10.50 (5.88, 15.13) | 6.69 (4.00, 9.38) | 4.21 (1.27, 7.15) | 4.50 (2.95, 6.06) | 3.00 (-0.75, 6.75) | **CT** |

**Supplementary Table S4 Results of network Meta-Analysis(LVEDD)**

| **CoQ_10_ 150mg** |  |  |  |  |
| --- | --- | --- | --- | --- |
| -0.61 (-3.64, 2.42) | **CoQ_10_ 90mg** |  |  |  |
| -3.10 (-7.64, 1.44) | -2.49 (-7.07, 2.09) | **n-3 PUFAs** |  |  |
| -2.98 (-5.67, -0.29) | -2.37 (-5.16, 0.41) | 0.12 (-3.93, 4.16) | **CoQ_10_ 30mg** |  |
| -5.40 (-8.14, -2.66) | -4.79 (-7.59, -1.99) | -2.30 (-5.92, 1.32) | -2.42 (-4.22, -0.62) | **CT** |

**Supplementary Table S5 Results of network Meta-Analysis(LVESD)**

| **CoQ_10_ 150mg** |  |  |  |  |
| --- | --- | --- | --- | --- |
| -2.12 (-4.72, 0.49) | **CoQ_10_ 90mg** |  |  |  |
| -2.41 (-6.38, 1.57) | -0.29 (-4.32, 3.74) | **n-3 PUFAs** |  |  |
| -3.79 (-6.08, -1.50) | -1.68 (-4.11, 0.76) | -1.39 (-5.05, 2.27) | **CoQ_10_ 30mg** |  |
| -6.31 (-8.60, -4.01) | -4.19 (-6.57, -1.81) | -3.90 (-7.15, -0.65) | -2.51 (-4.19, -0.83) | **CT** |

**Supplementary Table S6 Results of network Meta-Analysis(HR)**

| **CoQ10 60mg** |  |  |  |
| --- | --- | --- | --- |
| -3.06 (-9.58, 3.47) | **n-3 PUFAs** |  |  |
| -5.83 (-13.26, 1.60) | -2.77 (-6.68, 1.14) | **CoQ_10_ 90mg** |  |
| -7.00 (-13.42, -0.58) | -3.94 (-5.11, -2.77) | -1.17 (-4.90, 2.56) | **CT** |

**Supplementary Table S7 Pairwise meta-analysis (NT-proBNP, NYHA)**

| Analysis | Model | Estimate (95% CI) | I² | k |
| --- | --- | --- | --- | --- |
| NT-proBNP, CoQ_10_ vs CT (MD, pg/mL) | RE-REML | −264 (−447, −82) | 99.6% | 3 |
| NT-proBNP, CoQ_10_ vs CT — excl. confounded (MD) | RE-REML | −172 (−187, −157) | 0% | 2 |
| NYHA mean, CoQ_10_ vs CT (MD, class) | RE-REML | −0.41 (−0.60, −0.22) | 0% | 2 |
| NYHA improvement ≥1 class, CoQ_10_ vs CT (RR) | RE-REML | 1.23 (1.10, 1.38) | 0% | 5 |
| NYHA improvement, n-3 vs CT (RR, single) | single+cc | 36.5 (2.2, 592.8) | – | 1 |
| Exploratory: NYHA mean, combo vs CoQ_10_ 60 (MD) | single | −0.43 (−0.52, −0.34) | – | 1 |
| Exploratory: NYHA improvement, combo vs CoQ_10_ 60 (RR) | single | 1.23 (1.06, 1.42) | – | 1 |

**Note:** NT-proBNP and NYHA were analysed by conventional pairwise meta-analysis (not NMA). cc, continuity correction for a zero-event arm. RE-REML, random-effects restricted maximum likelihood.

**Supplementary Table S8. Subgroup analyses (supplement vs CT, besides LVEF)**

| Outcome | Subgroup | k | MD (95% CI) | I² |
| --- | --- | --- | --- | --- |
| LVEDD | Overall | 7 | −3.12 (−4.33, −1.91) | 99.7% |
| LVEDD | Duration: short-term (<3 mo) | 2 | −2.80 (−8.28, 2.68) | 75.2% |
| LVEDD | Duration: medium-term (3–<6 mo) | 4 | −3.20 (−4.71, −1.68) | 99.8% |
| LVEDD | Duration: long-term (≥6 mo) | 1 | −2.30 (−4.24, −0.36) | – |
| LVESD | Overall | 6 | −3.60 (−4.97, −2.23) | 82.2% |
| LVESD | Duration: short-term (<3 mo) | 1 | −1.40 (−6.18, 3.38) | – |
| LVESD | Duration: medium-term (3–<6 mo) | 4 | −3.71 (−5.46, −1.97) | 90.3% |
| LVESD | Duration: long-term (≥6 mo) | 1 | −3.90 (−6.18, −1.62) | – |

**Note:** Post-hoc subgroup pairwise meta-analyses (random-effects REML). k = number of supplement-vs-CT contrasts. Only trials with a CT/placebo comparator contribute; active-comparator trials (Azuma 1992, Wei YJ 2019, Yang YZ 2001) were excluded; multi-arm control groups were split across comparisons. Between-subgroup (interaction) tests for LVEF: duration *P* = 0.329, country *P* = 0.296, CoQ_10_ dose *P* < 0.001.

**Supplementary Table S9. GRADE certainty of evidence**

| Outcome | Comparison | Effect (95% CI) | Risk of bias | Inconsistency | Indirectness | Imprecision | Publication bias | Certainty | Key reasons |
| --- | --- | --- | --- | --- | --- | --- | --- | --- | --- |
| LVEF | n-3 PUFA vs CT | +4.21 (1.27, 7.15) | Not serious | Not serious | Not serious | Not serious | Suspected (−1) | MODERATE | Two double-blind RCTs; Nodari 2009/2011 |
| LVEF | CoQ_10_ 150 vs CT | +11.26 (8.55, 13.98) | Serious (−1) | Serious (−1) [global P = 0.065] | Not serious | Not serious | Suspected (−1) | VERY LOW | Single small trial (WangH 2018); implausibly small SDs for ventricular dimensions |
| LVEF | CoQ_10_ 90 vs CT | +6.69 (3.99, 9.38) | Serious (−1) | Serious (−1) [local P = 0.042] | Not serious | Not serious | Suspected (−1) | VERY LOW | Two small open-label Chinese trials (Xing 1999, WangH 2018) |
| LVEF | CoQ_10_ 30 vs CT | +4.50 (2.95, 6.06) | Serious (−1) | Not serious | Not serious | Not serious | Suspected (−1) | LOW | Robust after excluding all three confounded trials (Mei/levosimendan, Song/Qiliqiangxin, Dong/metoprolol): MD +4.18 (95% CI +2.52 to +5.85); the Dong CoQ_10_ 60 mg node was removed. |
| LVEF | CoQ_10_ 60 vs CT | +3.00 (−0.75, 6.75) | Serious (−1) | Not serious | Serious (−1) [co-administered metoprolol; β-blocker effect inseparable] | Serious (−1) [crosses null] | Suspected (−1) | VERY LOW | Single trial (Dong 2010); not significant; excluded in the co-intervention sensitivity analysis, which removed this node. |
| LVEF | Taurine vs CT | +10.50 (5.88, 15.13) | Serious (−1) | Not serious | Serious (−1) [connected to CT only indirectly via CoQ_10_ 30mg node] | Serious (−1) [n = 7 vs 10] | Suspected (−1) | VERY LOW | Azuma 1992 (n = 17); comparator was CoQ_10_ 30 mg, not CT; 8/17 ischaemic cardiomyopathy |
| LVEDD | CoQ_10_ 150 vs CT | −5.40 (−8.14, −2.66) | Serious (−1) | Not serious | Not serious | Serious (−1) [effect disappears after excluding small-SD trials] | Suspected (−1) | VERY LOW | Driven by single trial with implausibly small SDs (WangH 2018) |
| LVEDD | CoQ_10_ 30 vs CT | −2.42 (−4.22, −0.62) | Serious (−1) | Not serious | Not serious | Serious (−1) [non-significant after excluding small-SD trials: −2.80] | Suspected (−1) | VERY LOW | Fragile; small-SD trials drive the pooled effect |
| LVESD | CoQ_10_ 150 vs CT | −6.31 (−8.60, −4.01) | Serious (−1) | Serious (−1) [global P = 0.008] | Not serious | Serious (−1) [single-trial dependent] | Suspected (−1) | VERY LOW | Significant global inconsistency; driven by one trial with implausible SDs |
| LVESD | n-3 PUFA vs CT | −3.90 (−7.15, −0.65) | Not serious | Serious (−1) [global P = 0.008] | Not serious | Serious (−1) [CI approaches null] | Suspected (−1) | LOW | Single double-blind RCT (Nodari 2011); borderline effect |
| HR | n-3 PUFA vs CT | −3.94 (−5.11, −2.77) | Not serious | Not serious [star network, no loops] | Not serious | Not serious | Suspected (−1) | MODERATE | Two double-blind RCTs; after excluding Dong 2010, the MD remained −3.94 bpm (95% CI −5.11 to −2.77) and SUCRA increased to 96.0%. |
| HR | CoQ_10_ 60 vs CT | −7.00 (−13.42, −0.58) | Serious (−1) | Not applicable [single study] | Serious (−1) [co-administered metoprolol] | Serious (−1) [wide CI, single trial] | Suspected (−1) | VERY LOW | Dong 2010; metoprolol confound; node removed after exclusion in sensitivity analysis. |
| NT-proBNP (pairwise) | CoQ_10_ vs CT | −264 (−447, −82) I² = 99.6% | Serious (−1) | Serious (−2) [I² = 99.6%] | Not serious | Not serious | Suspected (−1) | VERY LOW | Extreme heterogeneity from one trial co-administering levosimendan; after exclusion: −172 (−187, −157), I² ≈ 0% |
| NYHA mean (pairwise) | CoQ_10_ vs CT | −0.41 (−0.60, −0.22) I² ≈ 0% | Serious (−1) | Not serious | Not serious | Serious (−1) [k = 2, small total N] | Suspected (−1) | VERY LOW | Only 2 trials (Bi 2012, Keogh 2003); Keogh SEM converted to SD |
| NYHA improve (pairwise) | CoQ_10_ vs CT | RR 1.23 (1.10, 1.38) I² ≈ 0% | Serious (−1) | Not serious | Not serious | Not serious | Suspected (−1) | LOW | Consistent across 5 trials; proportion improving ≥1 NYHA class |
| NYHA (exploratory) | Combo vs CoQ_10_ 60 | MD −0.43 / RR 1.23 | Serious (−2) [single open-label trial; multi-component formula] | Not applicable [single study] | Serious (−1) [CoQ_10_ + taurine + Astragalus + Shengmai; taurine effect inseparable] | Serious (−1) [single trial] | Suspected (−1) | VERY LOW | Yang YZ 2001; active comparator (CoQ_10_ 60 mg), not CT; exploratory only |

**Note:** Downgrading domains: Risk of bias, Inconsistency, Indirectness, Imprecision, and suspected Publication bias. Certainty levels: HIGH → MODERATE (↓1) → LOW (↓2) → VERY LOW (↓3+). GRADE Working Group grades of evidence: HIGH — very confident the true effect lies close to the estimate; MODERATE — the true effect is probably close to the estimate; LOW — the true effect may be substantially different; VERY LOW — the true effect is likely substantially different. I², heterogeneity statistic; k, number of studies; NMA, network meta-analysis; SD, standard deviation; CI, confidence interval.

**Supplementary Table S10. Adverse events by study and arm (detailed)**

| Study | Arm | N | Dizziness | GI discomfort | Cold extremities | Nausea | Palpitations | Other events | Follow-up |
| --- | --- | --- | --- | --- | --- | --- | --- | --- | --- |
| Dong ZB 2010 | CT | 33 | 2 (6.1%) | — | — | — | — | — | 3 months |
| Dong ZB 2010 | CoQ_10_ 60 mg + metoprolol | 33 | — | 2 (6.1%) | 1 (3.0%) | — | — | — | 3 months |
| WangH 2018 | CoQ_10_ 30 mg | 20 | — | — | — | — | — | None | 3 months |
| WangH 2018 | CoQ_10_ 90 mg | 20 | — | — | — | 2 (10%) | — | — | 3 months |
| WangH 2018 | CoQ_10_ 150 mg | 20 | — | — | — | 3 (15%) | 1 (5%) | — | 3 months |
| Keogh 2003 | Placebo | 19 | — | 1 (rash+nausea) | — | — | — | 1 dyspepsia → withdrawal | 3 months |
| Keogh 2003 | CoQ_10_ 150 mg | 19 | — | — | — | — | — | 1 somnolence → withdrawal; 1 carvedilol start → withdrawn | 3 months |
| Nodari 2009 | Placebo | 22 | — | — | — | — | — | 3 hospitalisations (2 VT, 1 bradycardia); 2 dropouts (1 lung cancer, 1 ICD) | 6 months |
| Nodari 2009 | n-3 PUFA | 22 | — | — | — | — | — | No major events; 1 withdrawal of consent | 6 months |
| Nodari 2011 | Placebo | 66 | — | — | — | — | — | 28 all-cause hospitalisations (42.4%); 20 HF hospitalisations (30.3%); 9 ICD shocks | 12 months |
| Nodari 2011 | n-3 PUFA | 67 | — | — | — | — | — | 18 all-cause hospitalisations (26.9%); 4 HF hospitalisations (5.9%); 2 ICD shocks | 12 months |

**Supplementary Table S11. 95% prediction intervals for the network meta-analyses (each treatment versus CT).**

| **Outcome** | **Comparison (vs CT)** | **MD** | **95% CI** | **95% PI** |
| --- | --- | --- | --- | --- |
| LVEF (%) | CoQ_10_ 150 mg | 11.26 | 8.55, 13.98 | 6.99, 15.54 |
|  | CoQ_10_ 90 mg | 6.69 | 4.00, 9.38 | 2.43, 10.94 |
|  | CoQ_10_ 60 mg | 3.00 | −0.75, 6.75 | −2.19, 8.19 |
|  | CoQ_10_ 30 mg | 4.50 | 2.95, 6.06 | 1.06, 7.95 |
|  | Taurine | 10.50 | 5.88, 15.13 | 4.47, 16.54 |
|  | n-3 PUFAs | 4.21 | 1.27, 7.15 | −0.25, 8.67 |
| LVEDD (mm) | CoQ_10_ 150 mg | −5.40 | −8.14, −2.66 | −11.22, 0.42 |
|  | CoQ_10_ 90 mg | −4.79 | −7.59, −1.99 | −10.67, 1.09 |
|  | CoQ_10_ 30 mg | −2.42 | −4.22, −0.62 | −7.45, 2.61 |
|  | n-3 PUFAs | −2.30 | −5.92, 1.32 | −9.02, 4.42 |
| LVESD (mm) | CoQ_10_ 150 mg | −6.31 | −8.60, −4.01 | −11.60, −1.01 |
|  | CoQ_10_ 90 mg | −4.19 | −6.57, −1.81 | −9.59, 1.21 |
|  | CoQ_10_ 30 mg | −2.51 | −4.19, −0.83 | −7.16, 2.14 |
|  | n-3 PUFAs | −3.90 | −7.15, −0.65 | −10.39, 2.59 |
| HR (bpm) | CoQ_10_ 60 mg | −7.00 | −13.42, −0.58 | −21.10, 7.10 |
|  | CoQ_10_ 90 mg | −1.17 | −4.90, 2.56 | −9.36, 7.02 |
|  | n-3 PUFAs | −3.94 | −5.11, −2.77 | −6.51, −1.38 |

**Note:** Raw adverse-event counts as reported; denominators, severity grading and follow-up were inconsistently reported (see main text). MD, mean difference (units as per outcome); CI, confidence interval; PI, prediction interval. $\text{PI = MD ± }\text{t}_{\text{k−2}}\text{ × }\sqrt{\text{τ}^{\text{2}}\text{ + }\left( \text{SE} \right)^{\text{2}}}$. Common between-study SD (τ): 1.33 (LVEF), 1.56 (LVEDD), 1.18 (LVESD), ≈0 (HR). Wide PIs—several including the null—reflect the small number of trials and between-study heterogeneity.

**Figure S1 Risk of bias assessment included in the study**

**Figure S2 LVEF NMA forest (vs CT and key contrasts).**

**Figure S3 LVEDD NMA forest.**

**Figure S4 LVESD NMA forest.**

**Figure S5 HR NMA forest.**

**Figure S6 LVEF-Subgroup by treatment duration.**

**Figure S7 LVEF-Subgroup by country.**

**Figure S8. LVEF-subgroup by CoQ_10_ dose.**

**Figure S9. LVEDD-subgroup by treatment duration.**

**Figure S10. LVESD-subgroup by treatment duration.**

**Figure S11. LVEF network excluding all three confounded CoQ_10_ trials.**

**Note:** For the LVEF sensitivity analysis, trials with an active co-intervention administered only to the intervention arm were excluded (Mei 2023, Song 2009, and Dong 2010).

**Figure S12 LVEDD network excluding small-SD trials.**

**Figure S13 LVESD network excluding small-SD trials.**

**Figure S14. HR network after exclusion of a trial with an intervention-only co-treatment.**

**Note:** For the HR sensitivity analysis, the trial with an intervention-only active co-treatment was excluded.

**Appendix S1 Search strategy**

**1. PubMed**

#1 "Dietary Supplements"[MeSH Terms]

#2 "dietary supplements"[Title/Abstract] OR "dietary supplement"[Title/Abstract] OR "supplements dietary"[Title/Abstract] OR "dietary supplementations"[Title/Abstract] OR "supplementations, dietary"[Title/Abstract] OR "food supplementations"[Title/Abstract] OR "food supplements"[Title/Abstract] OR "food supplement"[Title/Abstract] OR "supplement, food"[Title/Abstract] OR "supplements, food"[Title/Abstract] OR "Nutraceuticals"[Title/Abstract] OR "Nutraceutical"[Title/Abstract] OR "Nutriceuticals"[Title/Abstract] OR "Nutriceutical"[Title/Abstract] OR "Neutraceuticals"[Title/Abstract] OR "Neutraceutical"[Title/Abstract] OR "herbal supplements"[Title/Abstract] OR "herbal supplement"[Title/Abstract] OR "supplement, herbal"[Title/Abstract] OR "supplements, herbal"[Title/Abstract]

#3 #1 OR #2

#4 "Cardiomyopathy, Dilated"[MeSH Terms]

#5 "Cardiomyopathies, Dilated"[Title/Abstract] OR "Dilated Cardiomyopathy"[Title/Abstract] OR "Cardiomyopathy, Familial Idiopathic"[Title/Abstract] OR "Cardiomyopathies, Familial Idiopathic"[Title/Abstract] OR "Familial Idiopathic Cardiomyopathies"[Title/Abstract] OR "Familial Idiopathic Cardiomyopathy"[Title/Abstract] OR "Idiopathic Cardiomyopathies, Familial"[Title/Abstract] OR "Idiopathic Cardiomyopathy, Familial"[Title/Abstract] OR "Cardiomyopathy, Dilated, with Conduction Deffect1"[Title/Abstract] OR "Congestive Cardiomyopathy"[Title/Abstract] OR "Cardiomyopathies, Congestive"[Title/Abstract] OR "Congestive Cardiomyopathies"[Title/Abstract] OR "Cardiomyopathy, Congestive"[Title/Abstract] OR "Cardiomyopathy, Dilated, CMD1A"[Title/Abstract] OR "Cardiomyopathy, Dilated, LMNA"[Title/Abstract] OR "Cardiomyopathy, Dilated, Autosomal Recessive"[Title/Abstract] OR "Cardiomyopathy, Dilated, 1a"[Title/Abstract] OR "Cardiomyopathy, Dilated, With Conduction Defect 1"[Title/Abstract] OR "Cardiomyopathy, Idiopathic Dilated"[Title/Abstract] OR "Cardiomyopathies, Idiopathic Dilated"[Title/Abstract] OR "Dilated Cardiomyopathies, Idiopathic"[Title/Abstract] OR "Dilated Cardiomyopathy, Idiopathic"[Title/Abstract] OR "Idiopathic Dilated Cardiomyopathies"[Title/Abstract] OR "Idiopathic Dilated Cardiomyopathy"[Title/Abstract]

#6 #4 OR #5

#7 #3 AND #6

**2. Cochrane Library (CENTRAL)**

#1 MeSH descriptor: [Dietary Supplements] explode all trees

#2 Dietary Supplement OR Nutriceuticals OR Nutraceutical OR Neutraceuticals OR Nutraceuticals OR Nutriceutical OR Neutraceutical OR Supplement, Herbal OR Supplements, Herbal OR Herbal Supplements OR Herbal Supplement OR Dietary Supplementations OR Supplementations, Dietary OR Supplement, Food OR Dietary Supplement OR Food Supplement OR Food Supplementations OR Supplements, Dietary OR Food Supplements OR Supplements, Food

#3 #1 OR #2

#4 MeSH descriptor: [Cardiomyopathy, Dilated] explode all trees

#5 Cardiomyopathies, Dilated OR Dilated Cardiomyopathy, Idiopathic OR Cardiomyopathy, Dilated, 1a OR Cardiomyopathy, Dilated, LMNA OR Congestive Cardiomyopathy OR Cardiomyopathy, Dilated, CMD1A OR Congestive Cardiomyopathies OR Idiopathic Cardiomyopathies, Familial OR Cardiomyopathies, Familial Idiopathic OR Cardiomyopathies, Idiopathic Dilated OR Idiopathic Cardiomyopathy, Familial OR Dilated Cardiomyopathies, Idiopathic OR Cardiomyopathy, Dilated, Autosomal Recessive OR Cardiomyopathies, Congestive OR Cardiomyopathy, Dilated, with Conduction Deffect1 OR Cardiomyopathy, Familial Idiopathic OR Idiopathic Dilated Cardiomyopathy OR Idiopathic Dilated Cardiomyopathies OR Cardiomyopathy, Dilated, With Conduction Defect 1 OR Cardiomyopathy, Congestive OR Cardiomyopathy, Idiopathic Dilated OR Familial Idiopathic Cardiomyopathy OR Familial Idiopathic Cardiomyopathies OR Dilated Cardiomyopathies OR Cardiomyopathies, Dilated OR Dilated Cardiomyopathy

#6 #4 OR #5

#7 #3 AND #6

**3. Web of Science Core Collection**

#1 TS=(Dietary Supplements OR Supplements, Dietary OR Dietary Supplementations OR Supplementations, Dietary OR Food Supplementations OR Food Supplements OR Food Supplement OR Supplement, Food OR Supplements, Food OR Nutraceuticals OR Nutraceutical OR Nutriceuticals OR Nutriceutical OR Neutraceuticals OR Neutraceutical OR Herbal Supplements OR Herbal Supplement OR Supplement, Herbal OR Supplements, Herbal)

#2 TS=(Cardiomyopathies, Dilated OR Dilated Cardiomyopathy OR Cardiomyopathy, Familial Idiopathic OR Cardiomyopathies, Familial Idiopathic OR Familial Idiopathic Cardiomyopathies OR Familial Idiopathic Cardiomyopathy OR Idiopathic Cardiomyopathies, Familial OR Idiopathic Cardiomyopathy, Familial OR Cardiomyopathy, Dilated, with Conduction Deffect1 OR Congestive Cardiomyopathy OR Cardiomyopathies, Congestive OR Congestive Cardiomyopathies OR Cardiomyopathy, Congestive OR Cardiomyopathy, Dilated, CMD1A OR Cardiomyopathy, Dilated, LMNA OR Cardiomyopathy, Dilated, Autosomal Recessive OR Cardiomyopathy, Dilated, 1a OR Cardiomyopathy, Dilated, With Conduction Defect 1 OR Cardiomyopathy, Idiopathic Dilated OR Cardiomyopathies, Idiopathic Dilated OR Dilated Cardiomyopathies, Idiopathic OR Dilated Cardiomyopathy, Idiopathic OR Idiopathic Dilated Cardiomyopathies OR Idiopathic Dilated Cardiomyopathy)

#3 #1 AND #2

**4. Embase**

#1 'dietary supplement'/exp OR 'dietary supplement'

#2 'diet additive':ab,ti OR 'diet supplement':ab,ti OR 'dietary supplements':ab,ti OR 'food supplement':ab,ti OR 'supplementary diet':ab,ti OR 'dietary supplement':ab,ti

#3 #1 OR #2

#4 'dilated cardiomyopathy'/exp

#5 'cardiomyopathy, congestive':ab,ti OR 'cardiomyopathy, dilated':ab,ti OR 'congestive cardio-myopathy':ab,ti OR 'congestive cardiomyopathies':ab,ti OR 'congestive cardiomyopathy':ab,ti OR 'congestive cmp':ab,ti OR 'congestive dilated cardiomyopathy':ab,ti OR 'congestive myocardiopathies':ab,ti OR 'congestive myocardiopathy':ab,ti OR 'dilated cardio-myopathy':ab,ti OR 'dilated cardiomyopathies':ab,ti OR 'dilated cmp':ab,ti OR 'myocardiopathy, congestive':ab,ti OR 'dilated cardiomyopathy':ab,ti

#6 #4 OR #5

#7 #3 AND #6

**The following English-language translations reproduce the concepts, field structure, and Boolean logic of the Chinese search strategies originally executed in each database.**

**5. CNKI (China National Knowledge Infrastructure)**

English translation of the Chinese-language search strategy:

TKA=("dietary supplement" + "diet" + "dietary nutritional supplement" + "nutritional supplement" + "nutrition" + "food and beverages" + "food" + "nutritional health product" + "nutritional product" + "nutritional preparation" + "nutritional medicine" + "tonic medicine" + "pharmaceutical nutritional product" + "food supplement" + "dietary supply" + "prebiotic" + "beneficial microbiota" + "synbiotic") AND TKA=("dilated cardiomyopathy" + "dilated cardiomyopathy 1A" + "cardiomyopathy, dilated")

**6. Wanfang Data**

English translation of the Chinese-language search strategy:

Topic: ("dietary supplement" OR "diet" OR "dietary nutritional supplement" OR "nutritional supplement" OR "nutrition" OR "food and beverages" OR "food" OR "nutritional health product" OR "nutritional product" OR "nutritional preparation" OR "nutritional medicine" OR "tonic medicine" OR "pharmaceutical nutritional product" OR "food supplement" OR "dietary supply" OR "prebiotic" OR "beneficial microbiota" OR "synbiotic") AND Topic: ("dilated cardiomyopathy" OR "dilated cardiomyopathy 1A" OR "cardiomyopathy, dilated")

**7. VIP (CQVIP Chinese Science and Technology Periodicals)**

English translation of the Chinese-language search strategy:

(M=("dietary supplement" OR "diet" OR "dietary nutritional supplement" OR "nutritional supplement" OR "nutrition" OR "food and beverages" OR "food" OR "nutritional health product" OR "nutritional product" OR "nutritional preparation" OR "nutritional medicine" OR "tonic medicine" OR "pharmaceutical nutritional product" OR "food supplement" OR "dietary supply" OR "prebiotic" OR "beneficial microbiota" OR "synbiotic") OR K=("dietary supplement" OR "diet" OR "dietary nutritional supplement" OR "nutritional supplement" OR "nutrition" OR "food and beverages" OR "food" OR "nutritional health product" OR "nutritional product" OR "nutritional preparation" OR "nutritional medicine" OR "tonic medicine" OR "pharmaceutical nutritional product" OR "food supplement" OR "dietary supply" OR "prebiotic" OR "beneficial microbiota" OR "synbiotic")) AND (M=("dilated cardiomyopathy" OR "dilated cardiomyopathy 1A" OR "cardiomyopathy, dilated") OR K=("dilated cardiomyopathy" OR "dilated cardiomyopathy 1A" OR "cardiomyopathy, dilated"))

**8. SinoMed (China Biology Medicine)**

English translation of the Chinese-language search strategy:

Subject heading "Dietary Supplements" (including the narrower and entry terms "nutritional health product," "nutritional product," "nutritional preparation," "nutritional medicine," "tonic medicine," "pharmaceutical nutritional product," "food supplement," and "dietary supply") AND subject heading "Cardiomyopathy, Dilated" (including "congestive cardiomyopathy," "dilated cardiomyopathy 1A," "dilated cardiomyopathy," "cardiomyopathy, familial idiopathic," and "familial idiopathic cardiomyopathy").
